# Collaboration, Gender, and Leadership at the Minnesota Seaside Station, 1901–1907

**DOI:** 10.1007/s10739-022-09679-4

**Published:** 2022-07-04

**Authors:** Sally Gregory Kohlstedt

**Affiliations:** University of Minnesota – Twin Cities, Minneapolis, MN USA

**Keywords:** Seaside stations, Women in science, Gender, Botany, Midwestern science, Land grant universities, Josephine Tilden, Conway MacMillan, Qualified mentorship

## Abstract

Mentorship and collaboration necessarily shaped opportunities for women in science, especially in the late nineteenth century at rapidly expanding public co-educational universities. A few male faculty made space for women to establish their own research programs and professional identities. At the University of Minnesota, botanist Conway MacMillan, an ambitious young department chair, provided a qualified mentorship to Josephine Tilden. He encouraged her research on algae and relied on her to do departmental support tasks even as he persuaded the administration to move her from instructor to assistant professor in 1903. Resulting publications on Minnesota algae led her to look further west, first at Yellowstone National Park and then along the Pacific Northwest coast. After visiting a particularly productive littoral site on Vancouver Island, she suggested that they establish a Minnesota Seaside Station there. Over its seven years in operation under the Midwestern leaders, that location proved remarkably productive. At the remote site, the two operated within their typical but not inevitable gendered roles and deliberately defined their seaside station as unconventional. They expected participants to study productively and, at the same time, find imaginative ways to enjoy nature at a place far from urban amenities. Gendered expectations remained casual as participants moved both within and against them. This study investigates how, in the early twentieth century, the role and expectations of mentorship shifted as Tilden established her own independent research agenda. The Minnesota Seaside Station, in particular, proved significant in developing the leadership skills essential for her to pursue research in the Pacific region at a time when American expansionism and indigenous cooperation made sites accessible to academic researchers.

Given her naturalist’s attention for detail, Josephine Tilden (1869–1957) recalled that when she first saw “a great sandstone shelf in which boulders have ground innumerable cistern-like pot-holes, varying in size from mere tea cups to great wells 30 feet across and 20 or more in depth,” and laced with marine plants and animals, she quickly recognized its potential for research (Anon. 1937).^1^ The adventuresome botanist had recently completed a master’s degree in botany in 1897 at the University of Minnesota and found in algae a promising research topic gaining attention in North America. On her return from her research trip to the Northwestern coast of North America, which had included that remote and windswept site on the western edge of Vancouver Island in August 1898, she shared her excitement with her advisor, Conway MacMillan (1867–1929). Just three years later, in 1901, the two botanists, somewhat improbably, launched their seaside research station on the Pacific Coast literally a half-continent away from their Midwestern land grant university. By then the enterprising young explorer was an instructor, soon to be the first woman appointed as Assistant Professor on the science faculty at the University of Minnesota.

The pattern of collaboration often involves tiered responsibility, including what historians and sociologists identify as invisible labor (Shapin 1989; Star and Strauss 1999). In recent decades, scholars have revealed how regularly men in authoritative positions whose accomplishments were well recognized even as informal acknowledgment was accorded sparingly to wives and assistants, overshadowed participation by women in science, consciously or not (Rossiter 1982; Pycior 1996; Lykknes 2012). Such hidden contributions were often substantial. Naomi Oreskes demonstrated that women in the physical sciences typically followed the norms of objectivity in their scientific work but had their efforts obscured by gendered assumptions and by the reality that their efforts often lacked the drama and heroics of masculine colleagues (Oreskes 1996). Recognition of the issue of distorted attribution of scientific work and women’s participation, sometimes leadership, provides a backdrop for my investigation of the early career of Josephine Tilden.

Individual stories, however, are complex because women found ways to claim results even as they negotiated gender expectations involving an apparent subordinate role. Josephine Tilden’s experience suggests just how such calculated behavior occurred within what might be termed qualified mentorship. Mutual and sometimes complementary roles advanced the early careers of both MacMillan and Tilden at the turn of the century, even as they often adhered to dominant gender norms. The two worked in tandem to establish a survey publication and strong department, and, most significantly, they planned and managed a relatively short-lived seaside station on Vancouver Island in the first decade of the twentieth century (Bartlett 1989). MacMillan seems to have intentionally mentored Tilden on and beyond campus as she pursued independent research but, simultaneously, expected some degree of assistance in his own work, including helping with illustrations for his publications and taking on library responsibilities for the department—thus qualifying his support by additional expectations. In short, his was a qualified mentorship. Closer analysis of how they worked together resists any simple binary reliance on male and female tropes while clearly also reflecting gendered norms.

As Erika Milam and Robert Nye have pointed out, men were caught up in scientific masculinities, which, in turn, marginalized women in science, technology, and medicine (Milam and Nye 2017). Men, too, needed to understand and relate to expectations that, for some, required masculine self-fashioning and emphasized male bonding and assertive, visible professional advancement. MacMillan understood his personal reputation was linked to that of the new Department of Botany at the University of Minnesota as he joined male business clubs in the community and emphasized the rough and rugged challenges of fieldwork. Tilden subscribed to feminine conventions as she travelled with her mother or other female companions, took on supporting tasks like artistic illustration for her departmental colleagues, and rather quietly engaged in local women’s networks.^2^ However, both academics seem to have found a greater degree of informality and license when they established a frontier-facing site physically distanced from social conventions. The full participation of women, ranging from graduate students to teaching botanists, created a cadre who encouraged each other to pursue research and teaching activities (Rossiter 1982).^3^

## Scientific Careers at an Expanding Land Grant State University

**Fig. 1:**
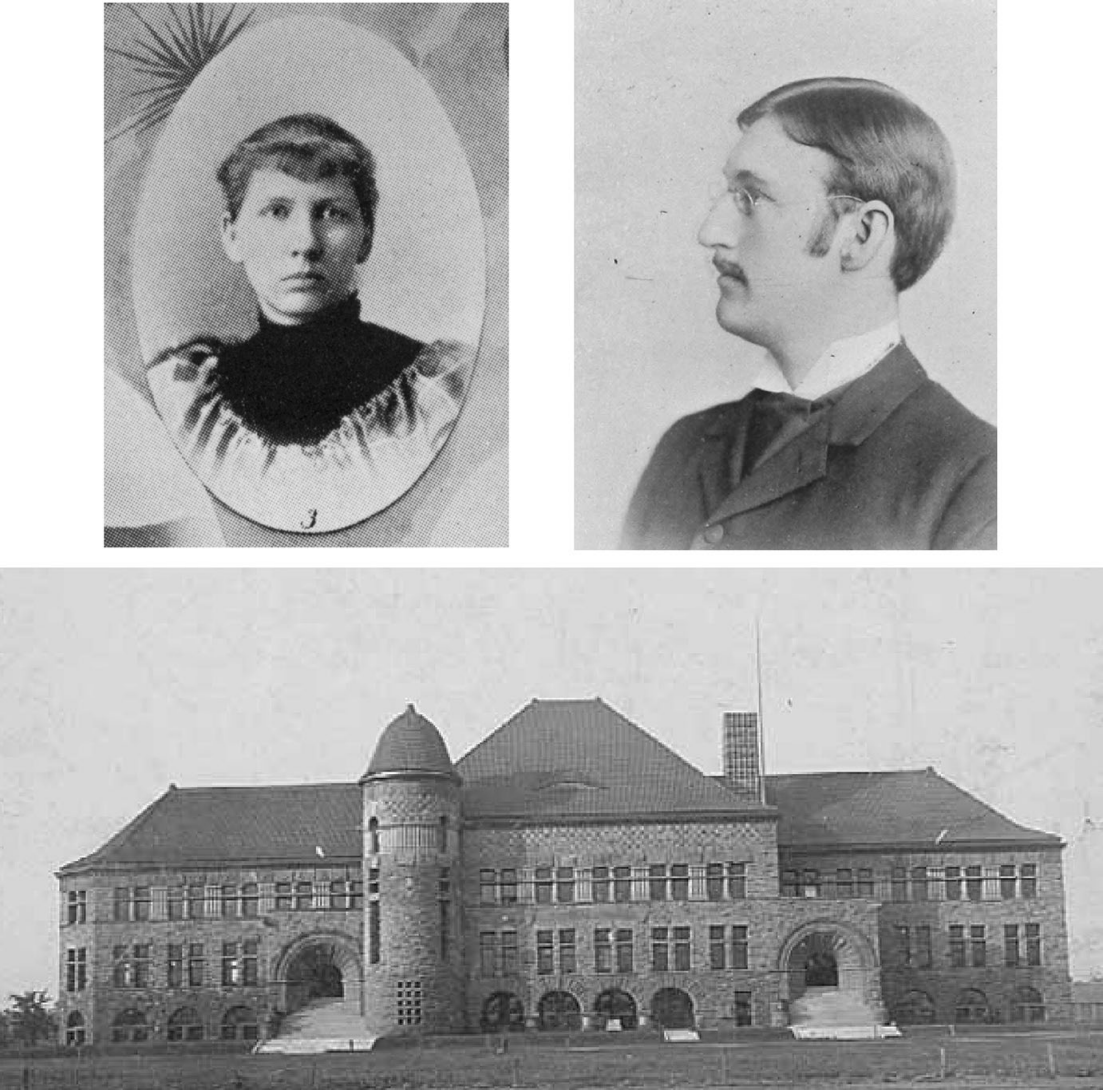
Josephine Tilden (top left) received her B.A. degree from the University of Minnesota in 1895. Conway MacMillan (top right) had joined the faculty in 1888 as professor of botany. Pillsbury Hall, opened in 1889, housed the Natural History Survey and held a growing herbarium on the second story of the right wing. *Gopher Yearbooks* for 1895 (p. 54) and 1897 (p. 103)

Tilden has attracted some historical attention based on her position as a “first,” but a thin archival record has limited close analysis of her personal life (Hanson 1918; Horsfield 2016; Moore and Toov 2015).^4^ She was the youngest of five children and only daughter, born in 1869 in Davenport, Iowa. Her father, a furnace handyman, had migrated west from upstate New York, married in Michigan, moved to Iowa, and then brought his wife and children to the growing and prosperous mill city of Minneapolis.^5^ At Central High School Tilden studied botany with Eloise Butler (1851–1933), a teacher and activist botanist who founded the Minneapolis Wildflower Garden (Hallender 1992, p. 49 ff.). Tilden entered the University of Minnesota in 1891 at the age of twenty, took Introductory Botany with Conway MacMillan, and soon contributed to the herbarium in newly built Pillsbury Hall. (Fig. 1) The ambitious instructor became head of Botany while still in his twenties, just as the university established specialized departments from its natural history curriculum.^6^ Nearly simultaneously appointed state botanist on the Minnesota Natural History Survey, MacMillan hired advanced students to assist him with plant collection and identification.^7^ State survey work provided important opportunities for scientific research at institutions designed to be primarily educational in the land grant act that provided their initial funds (Marcus 2015).

**Fig. 2:**
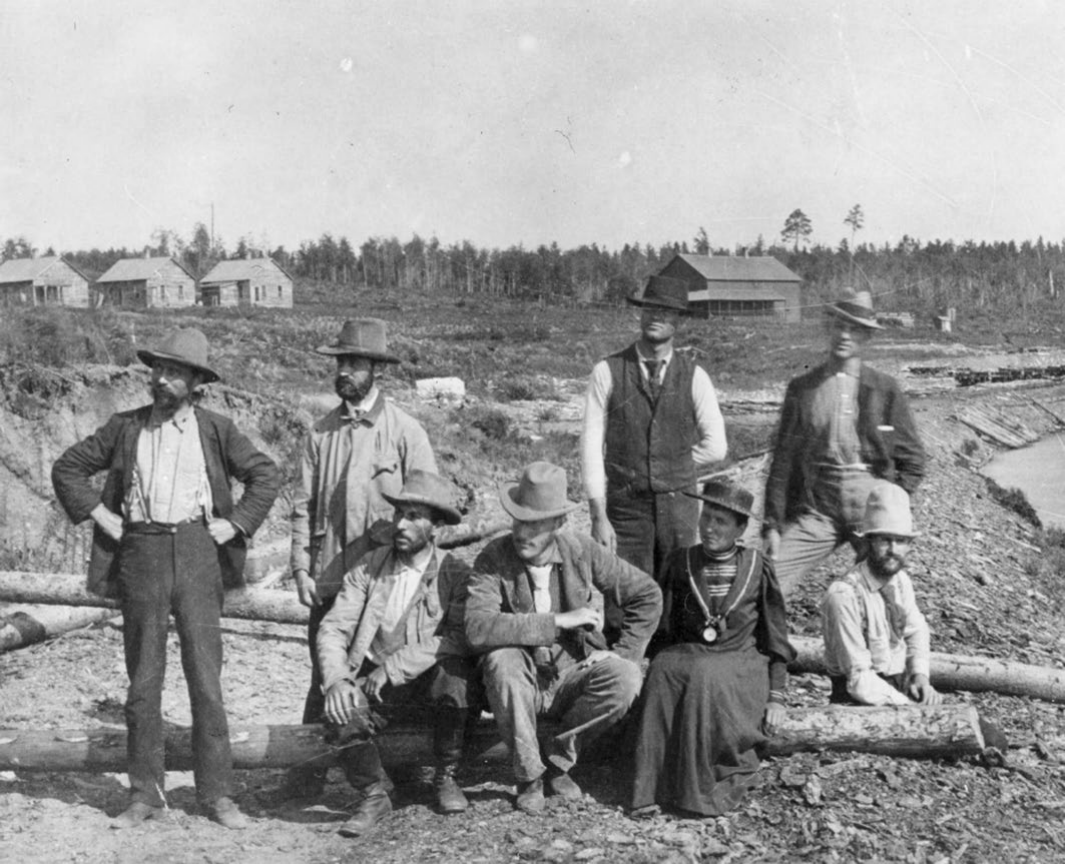
Professor Conway MacMillan took student Josephine Tilden and others to work for the summer on the Minnesota Geological and Natural History Survey in 1893. MacMillan is front and center, with Tilden seated on the log beside him at the site near Gull Lake. Zoology Department, UMNA

Tilden’s detailed class notebooks and collecting skills made the determined undergraduate stand out.^8^ She took up the topic of fresh water algae, filling a gap in expertise on the state survey, a topic neglected despite the evident opportunities available in a glaciated landscape with multiple lakes. This initiative earned her an appointment on the summer survey team based on Gull Lake, staying onsite with the otherwise all male team from June 16 to July 11, 1893.^9^ (Fig. 2) MacMillan optimistically hoped that this location with several worker’s cottages, placed at his disposal by the Northern Mill Company of Minneapolis, might become a permanent station; but there is no record of its continuing existence (MacMillan 1983).^10^

Women’s engagement with botanical studies had a long tradition. As Ann Shteir and others pointed out, the accessibility of such studies in girls’ academies in the early nineteenth century and subsequent opportunities to practice botany as illustrators and writers meant there were more women in botany than any other science. British historians observed that the availability of algae at summer recreational locations encouraged women to develop a special interest in seaside studies (Shteir 1996; Hunt 2005). Nonetheless, as Shteir, Margaret Rossiter, and others report, by the late nineteenth century there was pushback to women’s changing aspirations in botany that limited their access to resources and academic positions (Rossiter 1982; Chambers 2002; Tonn 2017). However, at new and formative institutions like the University of Minnesota that needed expert staff, exceptions could be made. While still an undergraduate, Tilden published two papers on algae. Clearly, she was exceptional.

Apparently living with her working parents, Tilden sought ways to earn money while in college. The rapid growth of botanical gardens and herbaria in the late nineteenth century created a market for closely defined systematic collections (Clements 1960, pp. 17–20).^11^ She discovered that creating a well-documented series of plants and producing an exsiccatae would certify her expertise and produce a modest income.^12^ Her first collection, one hundred specimens in an initial bound volume entitled *American Algae,* Century I*,* in 1894, featured Minnesota plants. She soon sold the dried and mounted algae with their careful botanical identification and locations of discovery to other collectors and herbaria.^13^ In the period of rapid growth of botanical gardens, herbaria, and academic departments, such projects earned income to the producer and simplified the study of the distribution of known species (Hung 2019; Kohlstedt 2021).^14^ The young Tilden was also keen to make new discoveries.

In the summer of 1894, still an undergraduate, she travelled with her mother to Yellowstone National Park, recently made accessible by the Great Northern Railroad with its local trunk line to the park.^15^ The working class young woman had the ongoing support of her mother as she followed prevailing gender norms for distant travel.^16^ Visiting its hot springs, Tilden studied the acidic and sometimes toxic blue-green algae that survived the fluctuating temperatures of the travertine formations and produced unusual algae stalactites (Tilden 1897). The park collections subsequently contributed to Century II and to her discovery of new red algae (Tilden 1897).

Taking her B.A. degree in 1895, she immediately started graduate studies and within two years had a master’s degree and an appointment as instructor. She supplemented her teaching income by serving as departmental librarian and continued to produce additional centuries of *American Algae*. This responsibility led her to begin a card index of publications on phycological literature and to make copies available to colleagues elsewhere.^17^ Tilden recognized that while Atlantic algae were increasingly documented, less attention had been given to marine plants on the Pacific Coast, especially in the American Northwest (Hallender 1992; Pastore 2021).^18^ The multivolume *Catalogue of Canadian Plants* by John Macoun (1831–1920) was largely completed by 1893. Its publication revealed not only what was known but also how much remained unknown, especially in the Canadian West (Macoun 1883–1902).^19^ Tentative plans to pursue advanced study abroad did not work out, perhaps for financial reasons, so Tilden stayed to teach at the University when the administration, via department chair MacMillan, promised her the books and sources she needed to investigate Pacific marine algae (Anon. 1937).^20^ Ambitious but with meagre resources, she struggled to pursue further studies and relied on the intervention of MacMillan as department chair to help negotiate research arrangements.

By contrast, Conway MacMillan had a privileged background and brought useful academic experiences to Minnesota. These enabled him to enter surprisingly quickly into academic life. His father was professor of Greek, initially at Hillsdale College in Hillsdale, Michigan, where MacMillan was in born in 1867. When his father took a position at the University of Nebraska, the younger MacMillan studied botany there with Charles Bessey.^21^ After his B.S. and then an M.A. degree in geology, MacMillan spent time at Johns Hopkins University.^22^ He later had an acrimonious, public exchange with faculty in its Department of Biology about the marginal status of botany there, and the tone points to his independent, occasionally combative, personality.^23^ He returned briefly to Nebraska to teach entomology in 1887 but then, probably on a recommendation from Charles Bessey, was offered a position in botany at Minnesota in 1888 (Frolick and Graham 1987, p. 217).^24^ He established contacts with well-established botanists, including Nathaniel Britton, soon to be director of the New York Botanical Garden, and initiated a Minnesota series of *Botanical Studies*, emulating the publication practices of professionalizing departments on the East Coast.^25^ Colleagues described MacMillan as “brilliant,” “erratic,” and with a genuine talent for writing.^26^ The confident young man also proved to be an effective speaker, called upon to talk about agricultural and botanical topics to local civic organizations and representing the university at the St. Louis World’s Fair.^27^ His connections and experience in the masculine world of higher education positioned him almost automatically in the field enterprise of survey work and station leadership (Milam and Nye 2017). Decades later, however, a friend from his Minnesota years reflected, without elaboration, that MacMillan seemed to have been an “early recognizer of the fact that women have an equal role in the professions.”^28^

The outlook in the Midwest at the turn of the century was optimistic, ambitious, and even assertive, as the region came of age in terms of economic, political, and even intellectual identity (Sisson et al. 2007, pp. 88–90). In these decades, Midwestern scientists far from the coasts were often self-conscious and aspirational as they pursued research and coordinated local and regional associations.^29^ That cultural conscious meant some of them, like MacMillan’s geologist colleague Newton Horace Winchell, maintained an edgy relationship with Eastern colleagues, feeling ignored or dismissed by those outside the professional circles established by location and collegiate degrees (Upham 1915). In the case of geology, this also reflected a degree of tension about expanding federal jurisdiction regarding scientific investigation (Bain 1916; Manning 1967, pp. 115–120).^30^

Both MacMillan and Tilden reflected a regional orientation as they built their personal careers and their department in an aspirational and rapidly growing land grant university. Their botanical work proved mutually productive in substantial ways even as their parallel lives reveal the possibilities and the limitations of gender, class, and professional networks.^31^ Particularly evident were subtle notions of masculinity that certainly echoed MacMillan’s experiences at male-dominated academic institutions like Harvard and Johns Hopkins and most scientific associations in that period (Bederman 1995; Rossiter 1982; Tonn 2017). The obviously talented and hardworking Tilden had few educational advantages, but she was able to marshal friends and students who contributed to her research. She also assumed tasks among her male botany colleagues that made her quite essential to their own work. Her efforts, as they recognized, contributed to the reputation of the botany department; indeed, she was among the most productive of its members. Nonetheless, MacMillan and other senior faculty in the college provided an essentially qualified mentorship to Tilden that differed from the support offered to junior men who joined the faculty during those years.^32^ Taking on independent projects was one way to demonstrate her talents and build a distinctive expertise.

## Venturing Further Afield

**Fig. 3:**
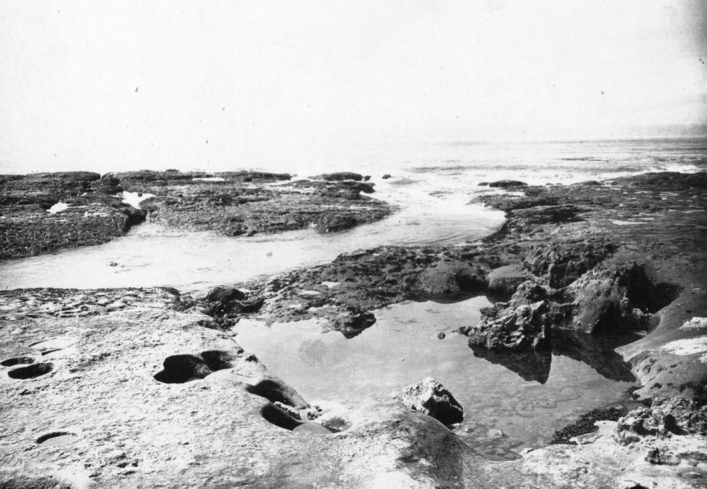
The rugged and irregular sandstone ledge was treacherous to ships at high tide but at low tide revealed a wealth of marine flora and fauna, some quite specific to this coast where the San Juan Strait meets the Pacific Ocean. Botany Department, UMNA

As she finished her master’s study, Tilden travelled to the Northwest in the late spring of 1897. She spent three months collecting algae on the American side of Puget Sound, traveling around Seattle, Tracyton, and the San Juan Islands, plus two weeks in northern California. Through local sea captains and fishermen in Puget Sound, Tilden learned about an active tidal region on the Pacific Coast reportedly treacherous for shipping that was simultaneously teeming with algae and sea life.^33^ The next summer, 1898, she spent 6 weeks in the San Juan Islands, then two months on the Canadian side of Puget Sound, at Oak Bay, Victoria, and finally ventured to the western coast of Vancouver Island. That trip required a ride on the weekly steamer to Port Renfrew on the San Juan River inlet, which offered a safe harbor along a coast that had been dubbed the Graveyard of the Pacific for its unpredictable weather and high tides.^34^ The small settlement had only a dock and small hotel, and from there she needed to travel either overland or find someone to take a small boat or canoe to the treacherous beach. Tilden soon discovered for herself why that stretch of the Pacific Coast was so notable (Fig. 3).

In an interview three decades later, Tilden recalled the drama as well as her intellectual excitement as she finally reached the site:

After a most disagreeable and terrifying trip on a small steamer [to Port Renfrew] my mother and I had a difficult time trying to find someone to row us over to a certain locality we had selected on the southern shore of Vancouver Island about 65 miles from Victoria, B.C. The United States Tide Table assured that at 4 pm on August fourth, the following day, there would occur the lowest tide that had happened in years. This spot which we were seeking had previously been described to me as the roughest spot on the shore of the Pacific Ocean by several sea captains…. We finally secured a gentleman named Tom Baird to transport us to the noted shore. On the way the waves drenched our boat and spoiled our food. But we were successful and the tide was lower that day than it has even been since. We remained four days with only a two-quart jar of cooked beans to eat and tea to drink. Wet to the skin and with no shelter from the rain, those four days were the happiest I have even spent. The algae covering that exposed shore … were beyond my wildest dreams. I spent every daylight moment in collecting algae. At stated intervals my mother doled out warmed up beans and tea. (Tilden 1937)^35^

The material in and around the tide pools provided Tilden with specimens for yet another exsiccatae of *American Algae*, Century IV, as well as adding to the botanical collections in the university’s museum.^36^ Baird, a local official intent on promoting development in the area, arranged for the intrepid woman to gain title to four acres of land adjoining the wild beach.^37^ Here was a place for potential new discoveries as well as a site with a wealth of algae specimens for classroom demonstrations.

Tilden returned to establish herself as an academic botanist. She was named Editorial Associate to a new monthly journal, *Plant World*, intended for botanical enthusiasts.^38^ She contributed an essay, “The Study of Algae,” to its first issue, in which she noted that this group included “the oldest, the lowest, the smallest, and the most widely distributed forms of plant life, and yet … they are perhaps the least understood and most generally unnoticed of any portion of the vegetable kingdom" (Tilden 1898, p. 148).^39^ The article went on to describe enthusiastically their adaptation to habitats as varied as the frozen oceans of the north to the boiling waters of geysers. She concluded her observations with the rather intimate suggestion that careful study of this species made it “impossible for one not to feel that plants are as much alive as himself" (Tilden 1898, p. 150).^40^ Her educational inclinations combined glimpses of a poetic sensibility with her scientific explanations of algae and their rich diversity, while she based her early research on detailed investigations relating to taxonomy and distribution of the species. During these years, Tilden continued to spend time between teaching periods investigating regional algae, writing shorter publications, and planning for an authoritative volume, *Minnesota Algae*, eventually published in 1910 (Tilden 1910).

Scientific curiosity and a desire to travel took her beyond the North American shore. Eager to learn more about Pacific algae, she persuaded her close friend, Caroline Crosby, to join her and her mother on a Hawaiian research expedition from May into July in the late spring of 1900. For nearly three months, they spent days exploring both freshwater and marine algae throughout the islands, with an emphasis on Oahu, in the territory recently annexed as United States territory in 1898. Crosby worked evenings with the microscope while Tilden prepared specimens that would contribute to additional volumes of *American Algae,* Centuries V and VI.^41^ This trip signalled Tilden’s growing interest in the distribution of algae in the greater Pacific region, looking toward the mid-ocean islands as well as the South Seas. Her aspirations were facilitated, indirectly, by the expansion of American political and economic influence that provided enhanced transportation and communication there in the early twentieth century.^42^

## Building Seaside Stations

The four days on Vancouver Island opened the possibility for a permanent station facing the Pacific. In the late nineteenth century, place-based research sites represented a new phase for biological research.^43^ In North America, agricultural and natural history survey research sites were typically linked to land grant universities and had a mandate for practical results.^44^ The Zoological Station in Naples, Italy, offered a different model and became significant for researchers engaged in marine biology, although Raf de Bont’s *Stations in the Field* describes how the idea of such stations also moved inland in Europe (de Bont 2015). By the early twentieth century, Americans identified promising locations by the sea and lakeshore as well as in deserts and mountains (Vetter 2012). An emphasis on biological research was the common factor because, as Robert Kohler points out, such stations had varying patterns of operation that used both laboratory and field practices shaped by the resources on each site and predilections of founders (Kohler 2012). Many of these sites, having originated with work on fishes, paid less attention to botany than to biology and physiology, even as they moved toward a more encompassing ecological outlook (Matlin 2020; Muka 2014a, b; Nyhart 2009). Keith Benson, Jane Maienschein, Frank Egerton, and others have demonstrated how the long-term seaside stations helped reshape natural history into ecological science and marine biology (Benson 1988, 2015; Maienschien 1989; Egerton 2014).

The multiple and diverse institutions provided uneven opportunities for women. Although Louis Agassiz’s Anderson School on Penikese Island in 1873 welcomed women teachers, as did its successor, the Marine Biological Laboratory at Woods Hole (Maienschien 1989), Margaret Rossiter points out that changing administration limited their leadership roles by a “rather brutal defeminization under the guise of higher standards” (Rossiter 1982, pp. 86–88; Burstyn 1977). Nonetheless, women able to participate found that, even marginalized, they had access to research facilities and professional networks that could advance their careers (Zottoli and Seyfarth 2015). Woods Hole also became famous for matchmaking, and some dual career couples met while studying there (Richmond 2012; Zottoli and Seyfarth 2015).^45^ As Jenna Tonn points out, women were rarely if ever among the leadership and their research results and aspirations could be made invisible by this pairing or sometimes overtaken by other life choices (Tonn 2019). Johns Hopkins laboratory in Beauford, North Carolina and most other stations affiliated with all-male universities proved reluctant (Wilder 1898; Allard 2000; Rudolph 1996). At Stanford University, which was a newcomer in higher education, coeducational, and engaged in preparing teachers of  nature study, David Starr Jordan and other administrators welcomed women to its Hopkins Seaside Laboratory on Monterey Bay, established in 1892.^46^ Simply by providing no living accommodations for women, stations like Carnegie Laboratory on Tortugas effectively excluded women altogether.^47^ Only decades later would a few exceptional women like Barbara McClintock gain permanent research appointments at facilities like Cold Spring Harbor (Keller 1983).

Given that few women who joined summer research programs held academic positions (aside from those at women’s colleges), most were teachers or graduate students. Tilden’s leadership, although not highly visible to outsiders, was thus distinctive. It is also worth noting that most seaside laboratories, even if in arguably isolated locations, had regular transportation and access to towns where provisions were readily available even as they sought sites presumed to be more natural (Schell 2017). A few provided significant amenities in terms of meals and accommodations, leading Phil Pauly to suggest that that Woods Hole, which had become a hub for marine research, had a club-like atmosphere that appealed to scientists “not interested in enduring the strenuous life” (Pauly 1988, pp. 121–122). While Woods Hole was better equipped than most, the Minnesota station was much more like a six-week expedition, with participants carrying what was required and, at some level, sustaining themselves by living off the land and sea.

The Midwestern academics, eager to demonstrate the scientific activities of their growing public university, named their Canadian site the Minnesota Seaside Station. Familiar with the East Coast stations, botanists MacMillan and Tilden intended their station to be a distinct version of the “place based” marine facilities and more broadly accessible. MacMillan advertised it as a “return to the simplicity and single-minded enthusiasm of the first American marine station, that at Penikese” (MacMillan 1902a). Given its remote, even primitive, location, the station emphasized marine botany and ecology rather than zoology and physiology, and thus reflected the research interests of its leaders, particularly Tilden. Her founding role invites attention to the ways in which both conventional and contrarian behaviors worked their way into this short-lived but effective seaside laboratory. The leaders shared the intention to facilitate original research and education found at other stations, even as they made clear in their announcements that their site on the edge of the Pacific Ocean offered new and distinct opportunities for discovery and research.

Having camped in rustic cabins in northern Minnesota with survey teams, Tilden and MacMillan had the imagination to envision something more rugged than established stations on the East Coast with their nearby amenities. Having gotten permission to build on the four-acre site offered by Baird,^48^ in the winter of 1900 MacMillan publicly announced their plan for the Minnesota Seaside Station, with a six-week session from July 15 to September 1, 1901 (Anon. 1901).^49^ The enthusiastic MacMillan told a Minnesota reporter that the Vancouver Island location was superior to those of stations on the Atlantic coast and went so far as to describe the others as “hackneyed, stale, and uninteresting in botanical lines when compared with the virgin shores of the north Pacific" (MacMillan 1901a, b). Typically, MacMillan took the lead in describing plans for the station and described himself as “director-in-chief.” Taking charge, coupled with his dynamic and visible leadership on site, projected a masculine stance even as he fully participated in the playful activities that relaxed the heroic masculine stereotype.

## Life and Leadership at the Minnesota Seaside Station

By January of 1901, twenty students and colleagues had signed on. The leaders had consulted widely, including with John Macoun, and had endorsements from the regional Canadian bishop, the mayor of Victoria, and other local officials. The University of Minnesota Board of Regents knew of the faculty-led project but resisted taking financial responsibility. MacMillan made it clear to the press that the university simply gave it administrative approval.^50^

Going to Port Renfrew in December of 1900, Tilden and MacMillan arranged with local settlers to construct two buildings. They planned rough but functional spaces that were sparsely furnished and able to house up to sixty persons in close quarters. Locals seemed intrigued by these unconventional newcomers and presumably anticipated this project would bring business and new residents. The larger two-story building had a large main room for study, socializing, and eating with a huge fireplace at one end. Its upper story housed a dormitory with balsam beds, in two rooms, one for men and one for women, thus enabling women to attend on an equal footing with men. There was also a small bedroom for Josephine Tilden and her mother. The smaller log cabin housed the laboratory. Here the participants found a dozen or so microscopes, reference books, as well as essential chemicals and glassware for their research and for preservation of specimens. Tilden designed the indoor spaces and began to arrange for transportation and supplies.^51^ The task was daunting because the rugged coastline did not allow for boats other than shallow canoes to land. A promised plank road from Port Renfrew was started but covered very little of the route, so attendees travelled three or more hours on foot over rough and often wet and slippery turf. One advantage of the deeply forested landscape was the onsite provision of wood for building.

While in Victoria, located on the southern end of Vancouver Island and the major port of British Columbia, MacMillan talked with a local reporter. He promoted the project and pointed out that the facilities were open to students of both sexes and that leaders hoped Canadian botanists would participate.^52^ As a confident and articulate spokesperson for the project, MacMillan organized publicity in local newspapers and scientific journals, especially the *Victoria Courier* and the *Minneapolis Tribune*. A University of Minnesota graduate teaching at Moorhead Normal School recalled that in those years MacMillan “showed the same power of organization and easy leadership which characterized his work elsewhere.”^53^ At the same time, Tilden began organizing for travel and the resources required for the summer work ahead.

With building plans in place, the instructors returned to Minnesota. Tilden booked a train car for participants from Minneapolis and St. Paul on the Canadian Pacific Railroad line through the scenic northern Rocky Mountains.^54^ Others from Wisconsin, Iowa, North and South Dakota, and Colorado arranged their own transportation, some using the Great Northern Line with connections to Seattle. The entire group needed to arrive at the same time in Victoria in order to catch the weekly steamer to Port Renfrew. The initial Minnesota group with Tilden took a leisurely trip of eight days as they enjoyed the scenery, gathered specimens, and took advantage of tourism sites, highlighted by the luxurious Banff Spring Hotel in Alberta, one of the early grand railway hotels. The cross-country trip, with its stops to study local flora and geology, was part of the attraction for the thirty-two Midwestern students and colleagues who attended the first year.^55^ Undergraduate Otto Rosendahl (1875–1966) took numerous photographs that commemorated stunning vistas of mountain passes and glaciers.^56^ The group also included a professional photographer from Minneapolis, C. J. Hibbard (1855–1924), who documented the Botanical Survey in Minnesota and produced hundreds of photographs of specimens along with landscape features.^57^ In Vancouver, the group found a town beginning to experience explosive population growth. From there they took a ferry to the well-established coastal city of Victoria where they enjoyed the amenities of civilization for one night in the well-appointed Dominion Hotel. Tilden coordinated the luggage and arranged for additional supplies. This stop included a tour of the local botanical garden before the group embarked on a five-hour turbulent ride on the weekly scheduled *Queen City* steamer to Port Renfrew, the small settlement safely tucked well into the Port San Juan inlet. Along the coast and inlets there were a number of native Pacheedaht summer encampments, whose members intermittently provided fresh fish to the academic visitors (Pacheedaht First Nation 2017).

Port Renfrew, with its recently settled white community, had a modest hotel and shop that served perhaps a dozen families on hardscrabble farms and a nearby lumbering operation. It was the first stop for the steamer going north out of Victoria along the western coast of Vancouver Island and thus the only source of mail and supplies (Scott 1974). The little settlement had a long dock, a hotel whose engaging Chinese cook was remarked upon by those anticipating camp fare ahead, but no real town yet.^58^ From there it was a three-mile overland hike, with luggage, to the botanical beach.

**Fig. 4:**
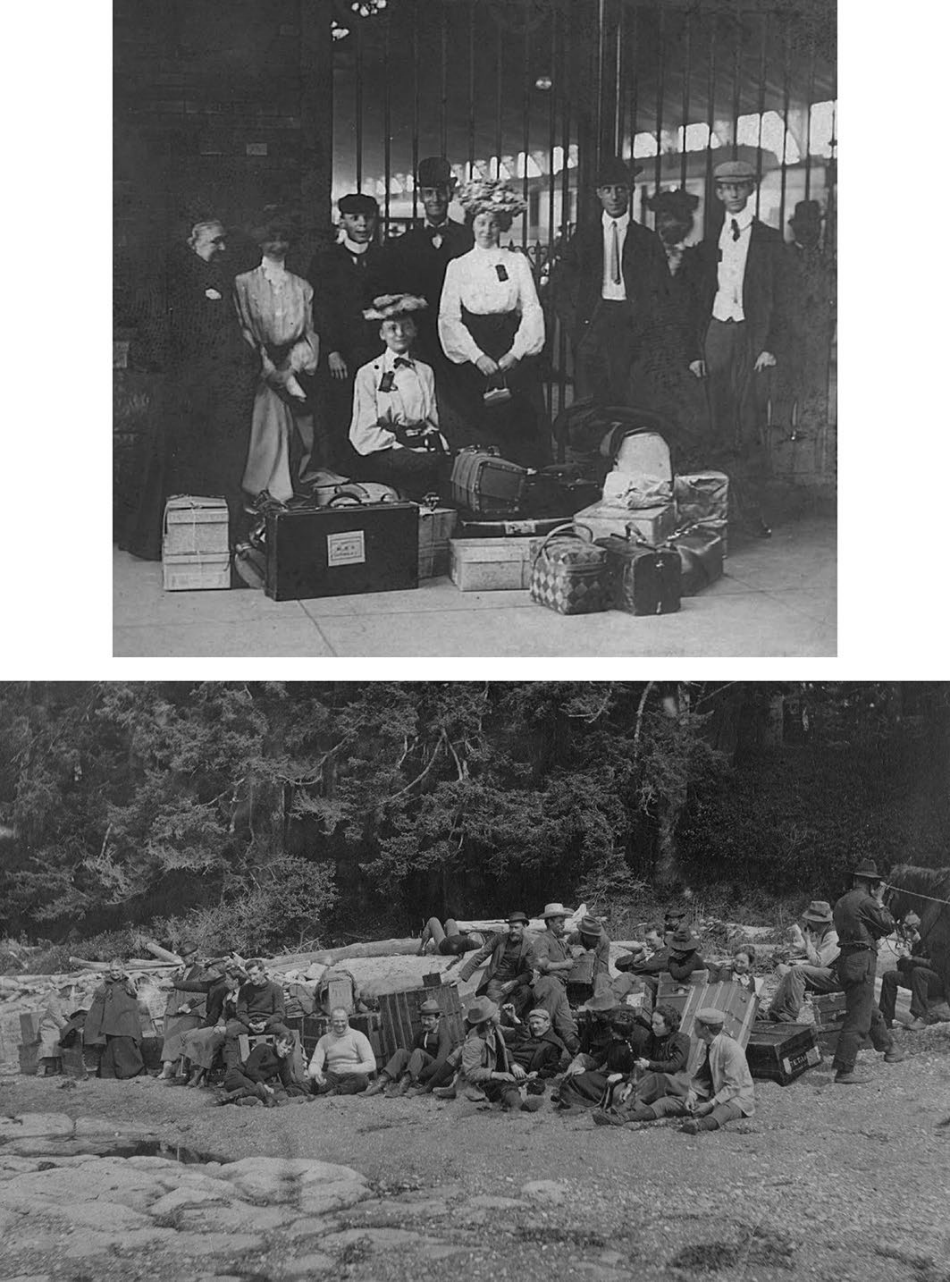
Formal dress was standard as a group assembled at the Minneapolis Union Station. Once at botanical beach, they assumed a more casual demeanor and dress. Rosendahl Photographs, Box 33, Folders 368 and 370, Botany Department, UMNA

While well-dressed travelers had gotten on the train in the Minneapolis, once on the Minnesota Seaside Station they needed to don more practical attire (Fig. 4). If the naturalists arrived at high tide, the windy surf could be dramatic and dangerous. The low tide revealed the extensive marine flora and fauna that had so caught Tilden’s imagination, but collecting them required careful footing when traversing the wet, slippery, and pockmarked beach. Here the scientific adventure became obvious.

Small notebooks with miscellaneous commentary containing grocery lists, packing notes, and names and addresses of contacts are part of recently recovered Tilden materials.^59^ They reveal how Tilden arranged for the travel, including booking tickets for the steamer from Victoria. She shipped the chemicals required for fixing specimens and brought boxes of glass jars and tin cans for specimen collecting, microscopes and books, paper for pressing plants, as well as groceries to supplement the diet that relied largely on local crustacean and fish.^60^ These housekeeping tasks relating to both domestic life and the academic activity at the station were simply assumed by Tilden. However, domestic roles never seemed to confine her, perhaps because she herself was strategic.^61^ She coordinated her own academic enterprise by marshalling her mother and all participants, male and female, in preparing group meals, gathering specimens, and collecting data that contributed to her teaching and publications.

Quietly committed to women’s rights, Tilden had actively recruited women among her students and friends, including her former teacher, Eloise Butler. She wanted them to be fully equipped to engage in the life of the station, including hiking and swimming as well as scientific work. An annual announcement was explicit about the gear that would make full participation possible: one pair of heavy-soled ten-inch- high bicycle shoes with hobnails (for hiking and climbing purposes), a bathing suit with high neck and long sleeves, and a “short skirt” hemmed twelve inches from the ground to deal with muddy, wet hikes. She also editorialized: “‘Good’ clothes are not even desirable since the work is rough, [so] one must be ready at all times of the day or evening for a tramp over the rocks or through the woods. Much of the restfulness comes from the absence of ‘competitive dressing’” (Anon. 1905). From the outset, women constituted roughly half of the participants, and they apparently enjoyed genuine camaraderie with the men in the group as investigators, although Tilden was the only woman instructor.

After facing the challenge of transporting luggage, including trunks with clothing and additional equipment, participants quickly discovered that hauling those items over the trail from Renfrew was nearly impossible. In subsequent years, the leaders alerted all participants to use satchels and pack lighter weight luggage rather than taking trunks.^62^ Clothing could be readily washed in one of the two streams that bordered the property, so taking multiples of similar dress was discouraged. Given the remote location, participants needed to arrive well prepared and self-sufficient in terms of supplies and equipment they might need as well as containers if they personally intended to bring back specimens for research and teaching. As Patience A. Schell points out, the deliberate isolation of many field science institutions inevitably required everyone to be attentive to the domestic aspects of life (Schell 2017).

Some scientific instructors, with MacMillan and Tilden in the lead, were relatively young but well equipped to examine nature from multiple perspectives. Raymond Osburn (1872–1955) of Ohio State University came to teach invertebrate zoology, while Minnesota’s Christopher W. Hall (1845–1911) taught geology. In 1901 and 1902, Kichisaburo Yendo (1871–1924), an advanced Japanese graduate student from the Imperial University of Tokyo studying algae, joined the teaching staff.^63^ He also introduced the group to the ways in which algae was prepared and used in Japan.^64^ Others with some expertise included Caswell Ballard from Moorhead State Normal School, Lawrence Waldron of North Dakota State at Fargo, and Lura Perrine of Valley City Normal School in Fargo. Family members came along, including MacMillan’s wife and daughter as well as Tilden’s mother and her friend Caroline Crosby. High school teachers and university students swelled the first attendance to thirty-five. The students, teachers, and faculty attendees, not unlike those initially at the Woods Hole and Stanford summer laboratories, were relatively young and eager to advance their careers.

## Patterns of Practice

While the social dynamics at the station were informal, the curriculum was apparently intense. Most participants were there to learn and gather materials for research or to take back with them for classroom use. Scheduled classes required flexibility because outdoor fieldwork was governed by tides and weather. High tides with strong winds could be dramatic, but their recession revealed an ever-changing site and offered the potential promise of new discoveries. Meals, too, relied on crustaceans, octopus, or small cod found hiding under ledges as the tide receded. It was a special meal when they caught or purchased salmon.

An undergraduate student, Alice Misz, wrote enthusiastic and detailed letters to her mother about life at the station in 1906. She noted, “I had class work all morning today, kelps and zoology, went to noon lecture and in the afternoon went to the beach and collected shells.” Reflecting on her seashore experience, she marvelled at the beauty of the wild, high waves. In that same letter, she informed her mother, “The work is much harder than I thought.”^65^ Another student reported:

If our tide table read ‘low-tide’ for five a.m., from half past four until breakfast time one might see about fifteen energetic students, armed with nets, knives, and pails, scattered about on the rocky shore. Several follow the receding tide, sliding over slippery kelp, splashing into small tide-pools, hidden by the treacherous ell-grass.… They pull aside heavy kelp leaves and disclose delicate seaweeds or tinted molluscs. (Janney 1904, p. 268)

**Fig. 5:**
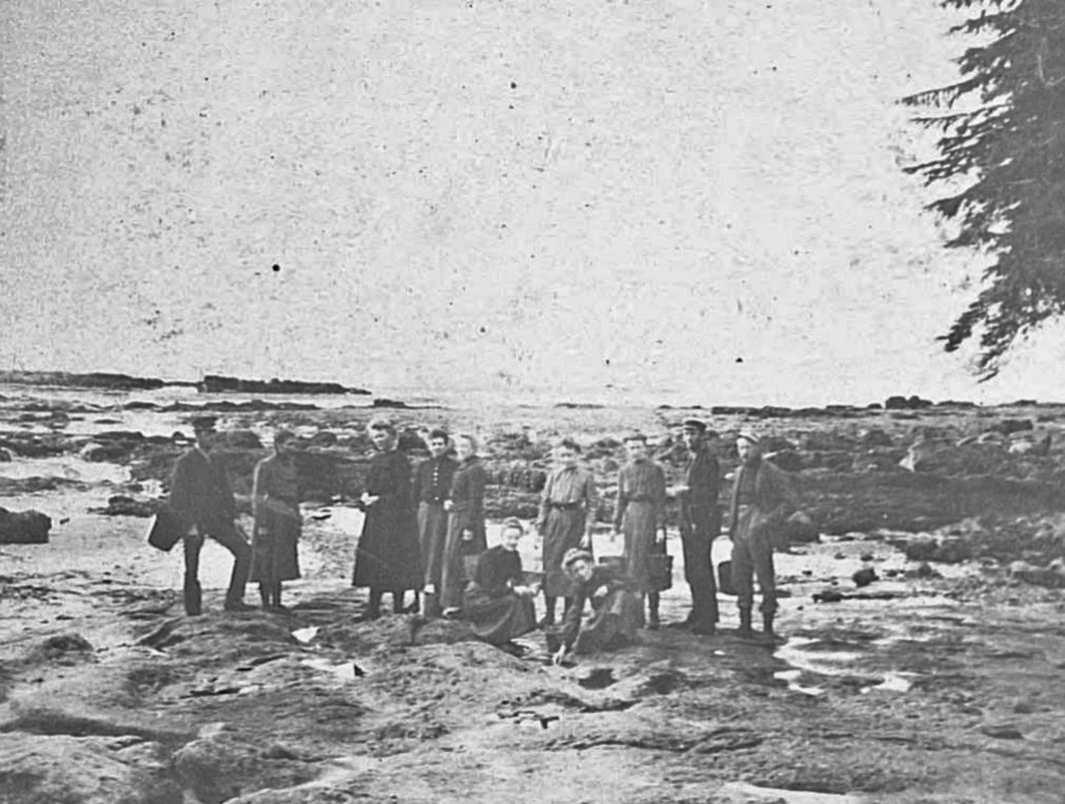
Josephine Tilden, kneeling at front right, posed with her class during an early morning low tide excursion to collect algae and sea life. Rosendahl Photographs, Box 33, Folder 368, Botany Department, UMNA

On such early mornings, the group worked several hours examining tide-pools, typically returning about eight o-clock for a well-earned breakfast.^66^ (Fig. 5) Participants had sufficient personal time to examine and reflect on the drama of the station with its ever-changing seascape, eroded shoreline, and extraordinarily old and distinctive lichens and trees that survived near the shore. Their commentary sometimes evoked the language of natural history, still visible and popular at the turn of the century, even as they relied on the technical insights of modern botany based on microscopic work (Kohler 2006; Kohlstedt 2010).

After classes and collecting expeditions, researchers spent late afternoons in the laboratories preparing specimens to ship back for detailed study and exchange. Most of the enrolled students were pursuing a master’s degree, and others attended classes but could take breaks to do their own exploration.^67^ Noon lectures—often called nature study—might be presented outdoors by an instructor of botany, zoology, or geology to illustrate how living things were an integral part of the specific and interconnected topography on the windswept side of Vancouver Island (Janney 1904, p. 268).^68^ Evenings were often casual, with fireside chats and reports of botanical excursions. Eloise Butler, Tilden’s former teacher, for example, gave an evening lecture about her botanical trip and algae collecting in Jamaica in the early 1890s (Butler 1902).

**Fig. 6:**
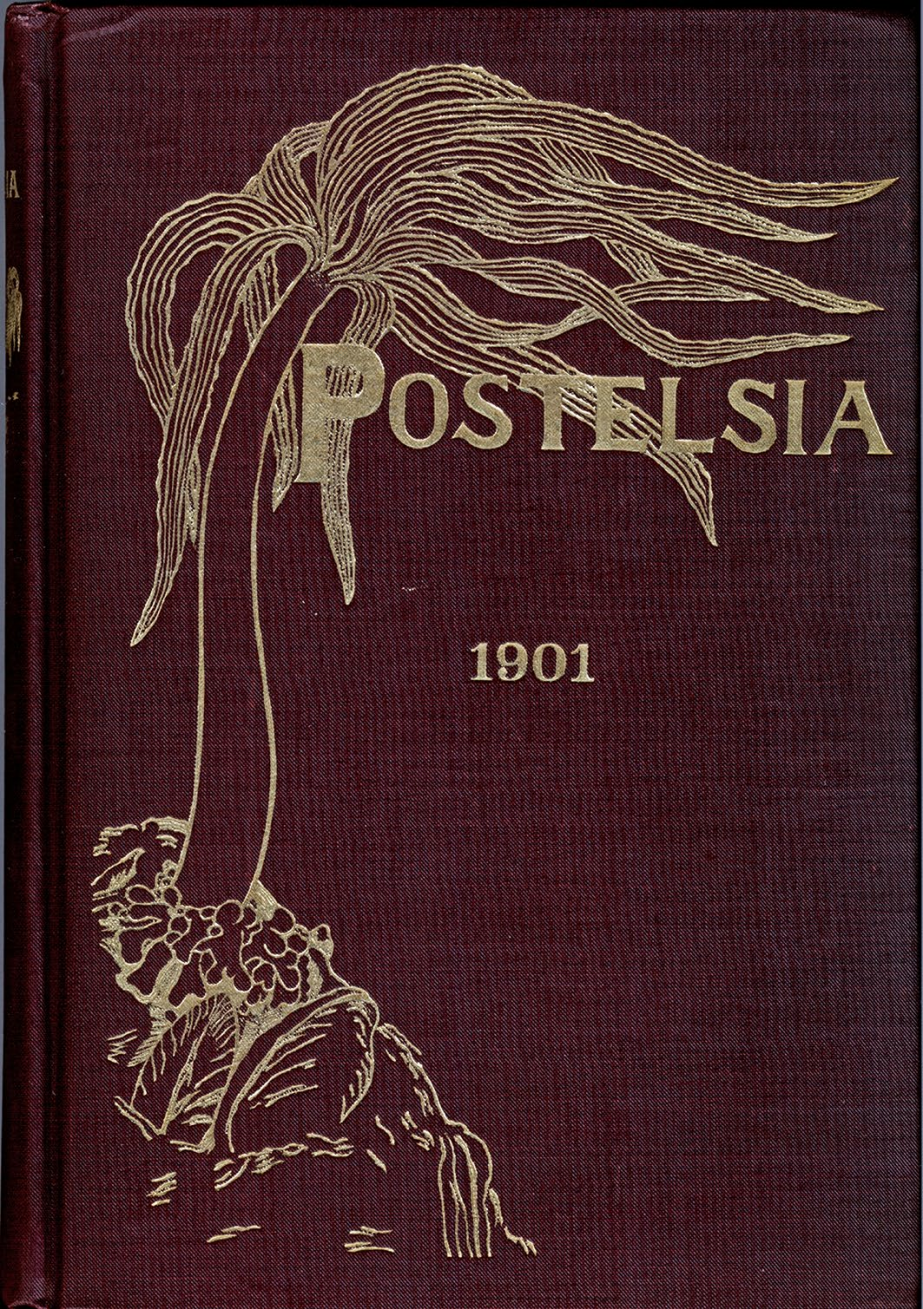
The large sea palm, *Postelsia*, became a symbol for the station, providing the title and cover for its initial publication (*Postelsia* 1901), a syllabus topic for campus class discussion and the name Postelsians for attendees. Photo courtesy of Rebecca Toor

Some of the midday and evening lectures, including that of Butler, were collected in an edited volume entitled *Postelsia,* the name of the large and distinctive sea palm that grew on a nearby point.^69^ (Fig. 6) Given the isolation of the wilderness station, the participants found creative ways to build a community culture, naming their buildings, establishing rituals, and creating plays that could be elaborated based on hand-written notes passed down from year to year.^70^ Informally, too, the attendees referred to themselves collectively as Postelians.

Instructors occasionally took classes further afield for study. With no local amenities nearby to break the routine, smaller groups organized strenuous two- and three-day excursions in the region. Some collected along the Gordon River on the other side of the San Juan inlet, while others joined geologist Hall to explore inland glaciers on Mount Edinburgh.^71^ These expeditions expanded the repertoire of students interested in distribution of land plants and in ecological relationships.

**Fig. 7:**
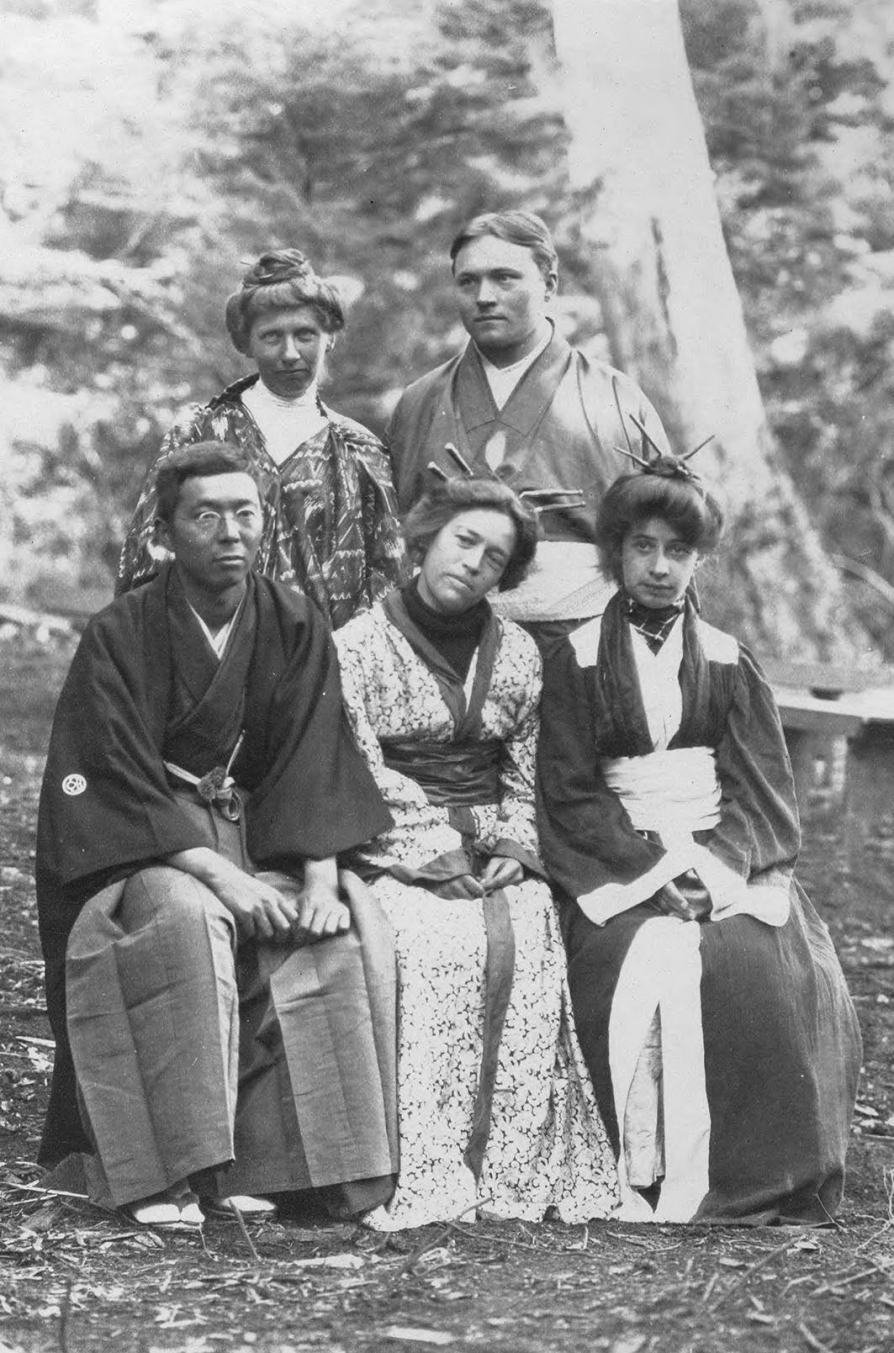
Dressed for a Japanese tea party in after hours  entertainment, Josephine Tilden is standing on the left side. Kichisaburo Yendo, their visiting instructor from the Imperial University in Tokyo, sat in front of her and next to Tilden’s close friend Caroline Crosby. Botany Department, UMNA

Weekends provided time for a break in routine and entertainment. The Japanese participant, Yendo, provided robes to augment a Japanese tea party. (Fig. 7) Other participants used unconventional or creative dress for amusement or practical purposes, sometimes cross-dressing, as when Conway put on a dress for an evening play or women students donned overalls for a collecting expedition. Never directly addressed, gender norms seemed to be held casually, as individuals playfully enjoyed each other’s company across traditional boundaries. They also frequently wrote about food. Special cakes were common as were homemade candies, the specialty of some students. Undergraduate Emily Janney wrote a lively account of the leisure-time activities, including swimming on warmer afternoons and acting out plays in the evening (Janney 1904).

**Fig. 8:**
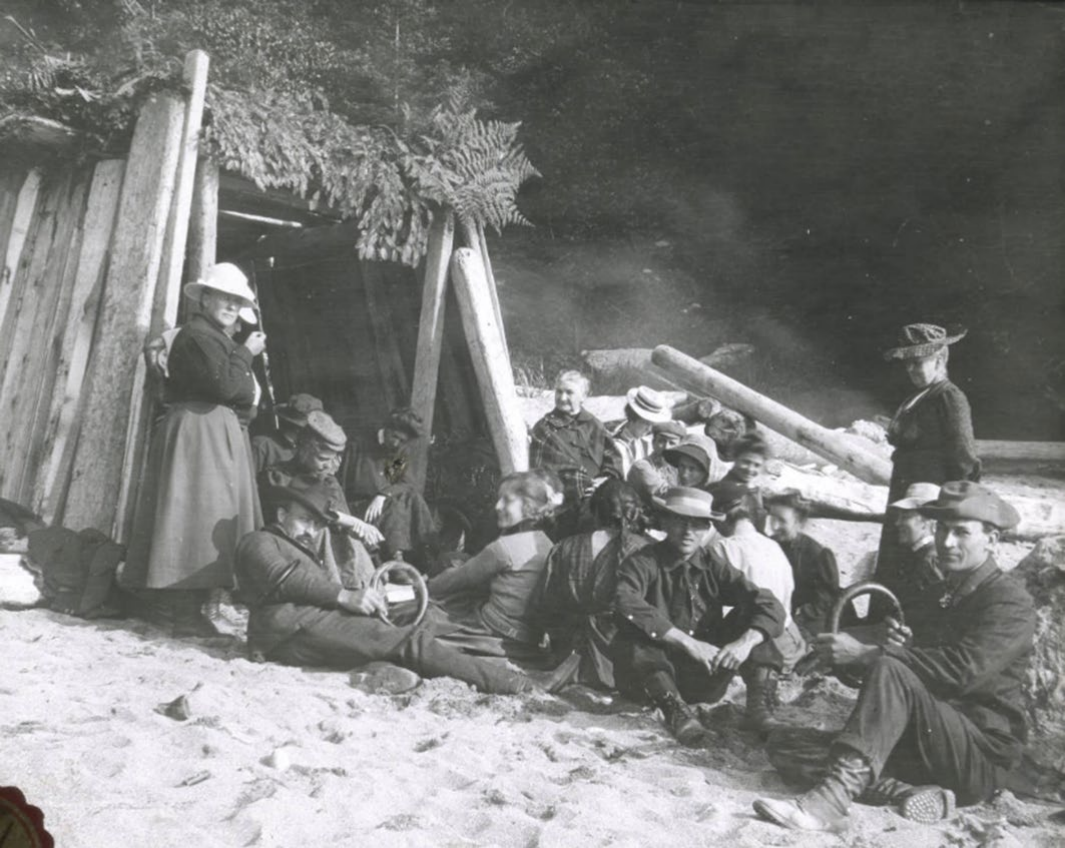
Some evenings particpants gathered on the beach to take advantage of make-shift huts (here the Griggs cottage) of driftwood and planks, which served to give couples and small groups more space and privacy than their dormitory accommodations. Ned Huff Photograph, Botany Department, UMNA

Younger married couples created some degree of privacy by building driftwood “campsites” on the beach and inviting others to join them. (Fig. 8) In good weather, evenings might be spent around roaring outdoor campfires where Postelians enjoyed the catch of the day, sometimes salmon from the San Juan inlet bought from indigenous people, locally called Siwash (now identified as Pacheedaht) who had a village further up the coast. Indeed, photographs reveal an interest in and exchanges with these neighbors who brought fish, sold their baskets, and occasionally transported the scientists in their dugout canoes as they engaged in the local settler economy.^72^ Tilden showed a genuine interest in the history of the region, including these indigenous people, an outlook echoed during years when she studied the native culture on Tahiti.^73^ At the station, she produced a historical play for the group with a series of scenes that followed human life on western Vancouver Island starting with the Siwash people and including subsequent landings by Spanish explorers, visits by pirates, sustained habitation by Dutch and English settlers, and finally the arrival of “Miss Tilden and botanists.”^74^

**Fig. 9:**
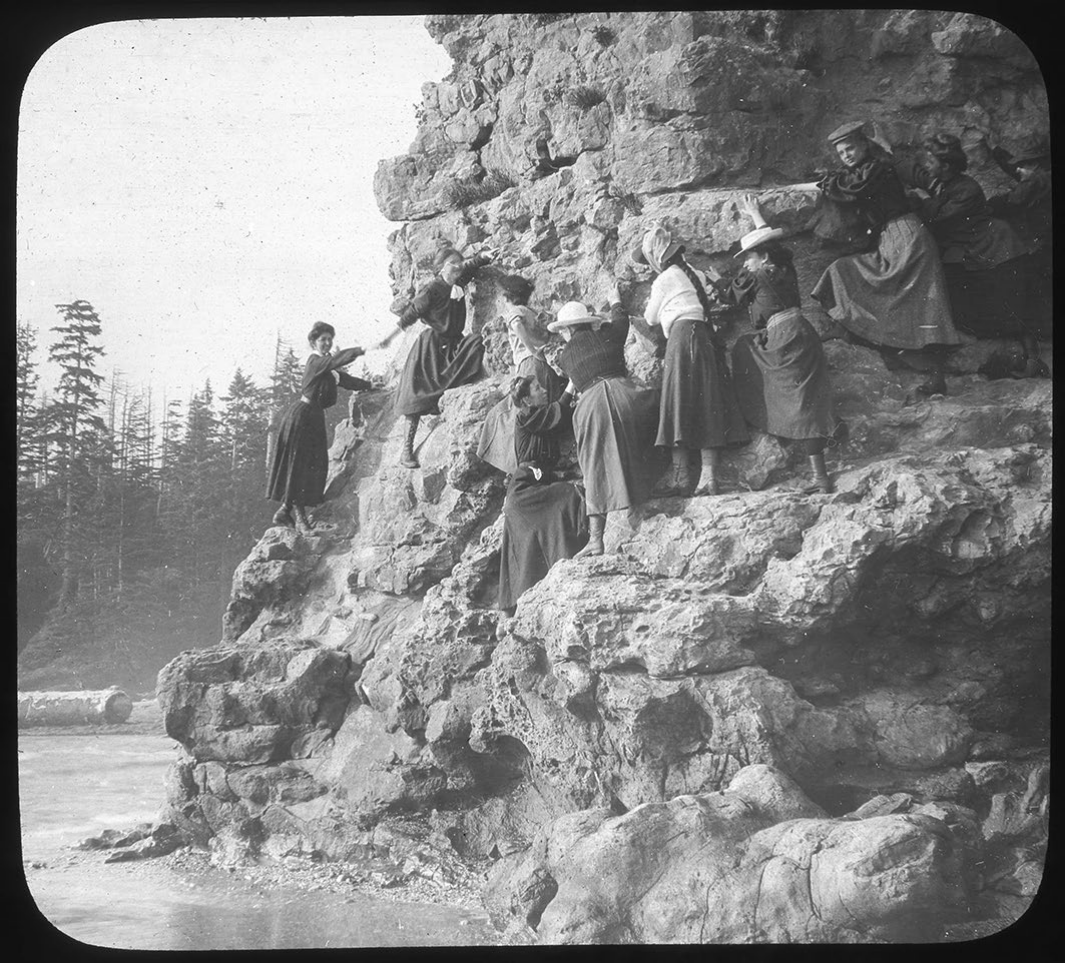
Some women posed at low tide on a rugged outcrop along one side of a massive cave dubbed “Hall of Energids” and demonstrated their working attire of shortened skirts and hobnailed boots. Ned Huff Lantern Slides, Botany Department, UMNA

**Fig. 10:**
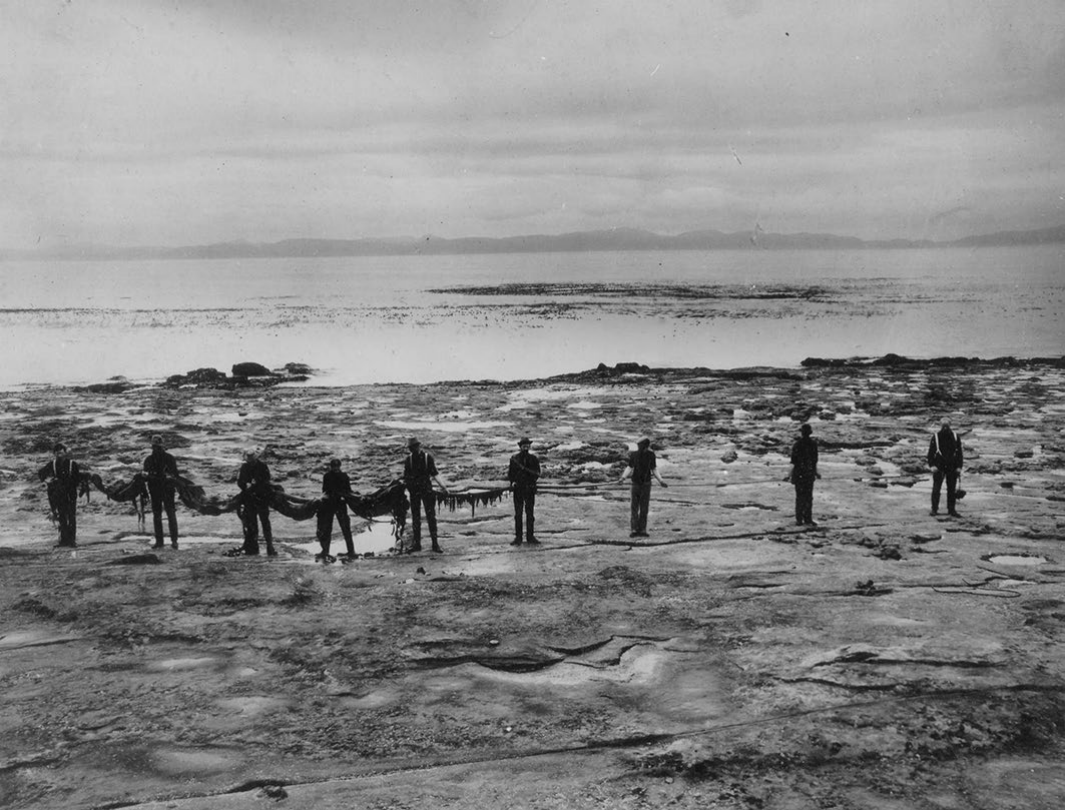
Participants were impressed with the large kelp strings, sometimes as long as forty feet in length; several men posed here with a singular heavy algae in the late afternoon. Botany Department, UMNA

The photographers in the group sought to capture moments of adventure each summer. A rugged rock outcrop on the edge of a cave designated “Hall of the Energids” provided a photographic opportunity, most dramatic when the tide came in. Here the women, wearing their hobnailed boots and skirts strategically hemmed twelve inches above the ground, posed to demonstrate their rock-climbing skills. (Fig. 9) Some of the men saw another opportunity for drama and posed with a forty-foot kelp. (Fig. 10) The group created camaraderie through humor and adventure as well as scientific collaboration, and Tilden reminiscenced about the “pure fun” they had when they took a break from intensive work.^75^

Borrowing from a popular Wisconsin myth about a mysterious Hodag, circulated in hunting and camping lore, the station held annual events around this elusive, reportedly dangerous creature (Kearney 1928). The entire group participated in a play whose choreographed script had participants seeking to propitiate the Hodag’s impending curse through offerings of maidens, providing exotic clothing, and presenting special foods. They conducted the play after dark to heighten the suspense.

With the 1901 summer session a decided success, the seaside laboratory advertised future summer sessions. Tilden returned in December to make further plans and to collect algae that would allow research on seasonal comparisons by her colleagues as well.^76^ The new announcements emphasized the opportunities for students and faculty to identify new species and varieties of seaweed, while teachers could acquire pressed plants and cans of specimens for their classrooms in the fall. MacMillan wrote an illustrated account for the winter issue of *Popular Science Monthly* to recruit participants for 1902. Acknowledging that the Minnesota Seaside Station was among the “youngest” of the seaside laboratories, he emphasized that it complemented the others with something “fresh” in its remoteness (MacMillan 1902b). Moreover, the cross-country train trip with its dramatic vistas provided opportunities to explore new geological and botanical landscapes before reaching the “stern and rock-bound coast.”

That coastal site indeed offered exploration and drama. Clear days provided a view across the Strait of Fuca to the Olympic Mountains. MacMillan pointed to the possibilities for ecological studies. Not only were zonal distributions important but the site also offered “sharp lines of demarcation between different algal societies” based on tidal action. His article is nearly lyrical in its discussion of just how the flora and fauna interacted on the exposed reefs by comparison with the tide pools where water movement is less violent. MacMillan pictured the possibilities of what he termed a “biological pilgrimage” for naturalists in the central-western states seeking pleasant and profitable weeks on the shore. At the same time he began to envision the modest camp developing into a “genuine marine laboratory with the full equipment and a field of usefulness peculiarly its own" (MacMillan 1902b, p. 208). He urged his former teacher, Charles Bessey, to note in *Science* that there were now two West Coast summer schools for botanical study, one at Pacific Grove and the other at Port Renfrew. The senior botanist made it clear that the two sites were not yet equivalent but generously observed both did work of high order.^77^

**Fig. 11:**
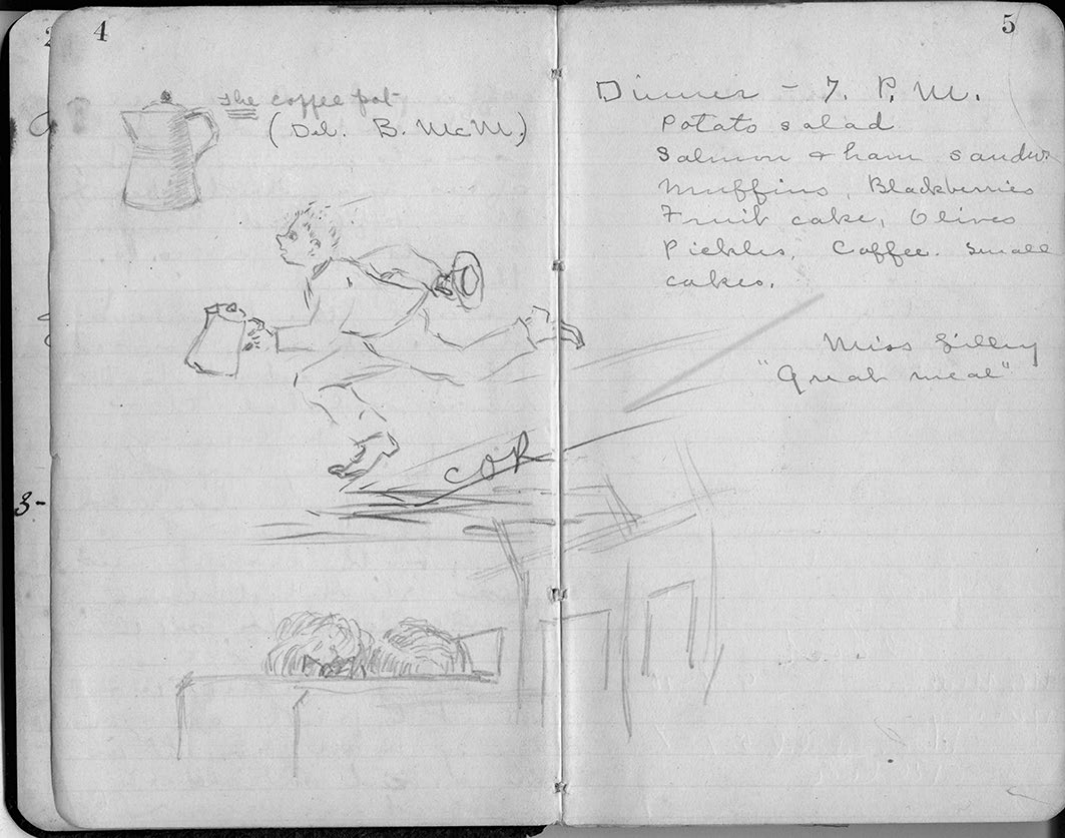
Tilden’s small notebooks held a variety of information, including occasional drawings as well as commentary on food and activities. Here she sketched the young man who brought the coffee pot (essential for the meals the group prepared) to their Pullman car headed to Vancouver; the dinner menu is in the upper right. “C” Notebook, Tilden Papers, Box 3, UMNA

Whether or not MacMillan and others acknowledged her role in print, Tilden remained central to the project and an essential part of its dynamism and humor. A small black notebook recorded how she organized eleven people to ride together in a separate train car so they could do some of their own meal preparation. A sketch also offered a rare glimpse of her humor. One colleague realized that there was no coffee pot, so as the train pulled through the small town of Hoffman, Tilden reported, “We all howled out the window for a coffee pot. A small freckle-faced boy came to the window and they offered him $.50 to fetch one. Just as the train pulled out, he came running alongside and they got their coffee pot.”^78^ (Fig. 11) At longer stops, the botanists left their railcar to identify flowers, others took photographs, and at one train stop a graduate student shot a duck for dinner. On site, Tilden also took a strong hand in organizing the daily agenda. Class schedules varied from year to year, based on experience and the particular mix of participants. In the early years, the norm seemed to be botany classes in the morning, a lunchtime lecture, and zoology lectures (marine animals and entomology) in the afternoons, with evening lectures or entertainment.^79^ She initially established a regular cooking rotation, which required all participants, men and women in teams, preparing meals each day. By 1903 there were distinct classes for high school teachers and for more advanced students, a recognition that cohorts had different levels of expertise and quite specific purposes for attending the summer program (Anon. 1903). The high school and normal school instructors were typically there to collect a variety of specimens for teaching, while the graduate students sought specific collections for papers and theses.

After two years, the station seemed to achieve a stable attendance with established routines that encouraged construction of a third, larger building designed for a botany lab, with the older log cabin redesignated for zoology. With so much progress, MacMillan was irritated when Charles Davenport of Cold Spring Harbor reported to *Science* magazine that there should be more marine laboratories on the West Coast and recommended Puget Sound as a potential site. Perhaps defensive about his Midwestern location, MacMillan presumed that Davenport was deliberately ignoring the new station at the Straits of Fuca that was already positioned to study the Sound as well as the open sea. MacMillan responded in the next issue of *Science* that while no millionaire had sponsored the Vancouver station (a somewhat sarcastic reference to Andrew Carnegie’s sponsorship of Cold Spring Harbor), it was nonetheless a high functioning and cooperative enterprise doing productive work.^80^ Having advertised the station widely, MacMillan interpreted the apparently deliberate oversight as insulting, although his own earlier advertising had disparaged the East Coast stations.^81^ Nonetheless, ever eager to promote the Minnesota site, he made the comparison work for him, explaining in his 1903 brochure, “the life is that of a camp, and is very novel and interesting to town and city bred people. There is a freedom and unconventionality which cannot be found in any of the Eastern laboratories" (Pelley 1985). He thus linked the Canadian-based station to other established stations on the other side of the continent even as he argued for his site’s distinctive configuration.^82^

**Fig. 12:**
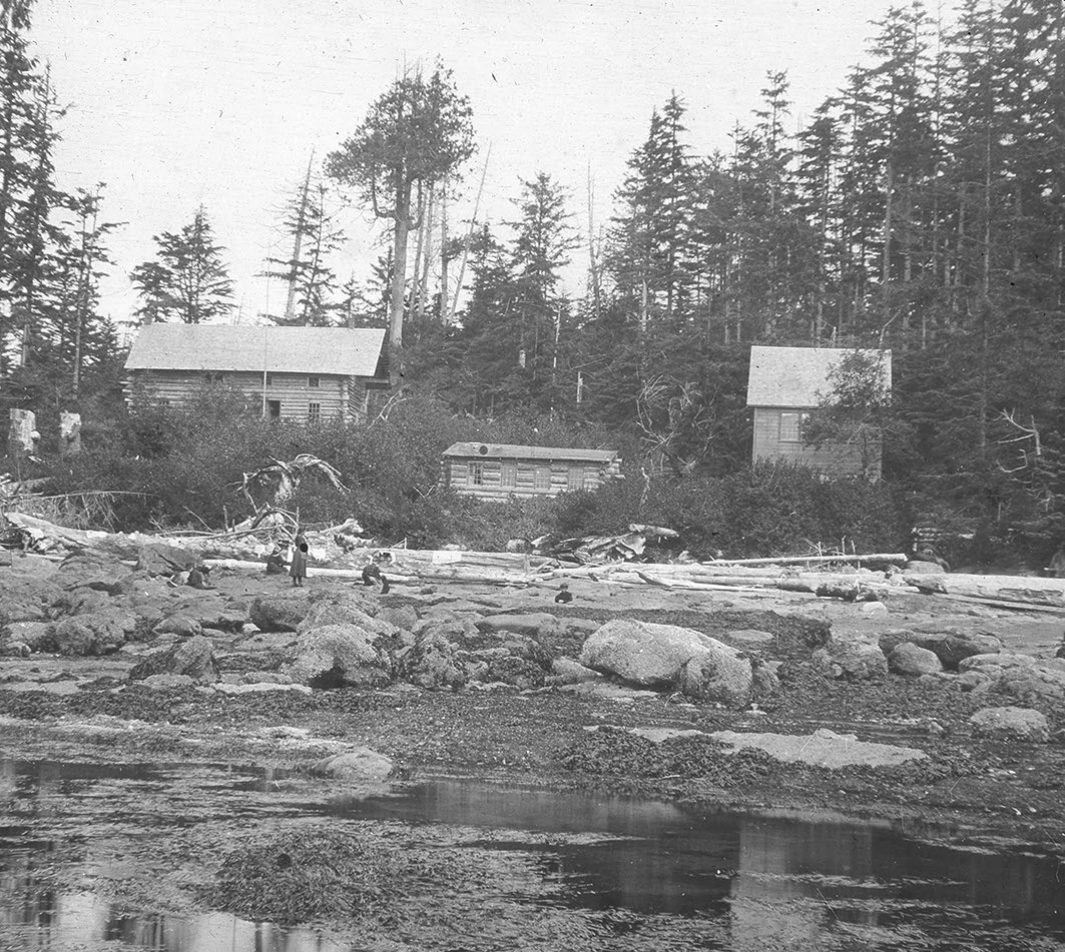
By 1904 the Minnesota Seaside Station had a main building with dormitory, botanical laboratory, and zoological working space. Here, too, students had easy access to the sandstone ledges immediately in front of the station at low tide. Ned Huff Photograph, Botany Department, UMNA

Still seeking ways to make the station more visible, in 1904 MacMillan invited his former professor at Nebraska, Charles Bessey, to spend time at the station. The well-known botanist subsequently wrote a lively account of his stay in *Popular Science Monthly.* He endorsed the project again and, as a participant observer, he commented enthusiastically that the giant cedar and fir trees, oversized ferns, and dramatic landscape provided new insights for an inland botanist like himself (Bessey 1905). He described his former student MacMillan as a “tall, stout man with a twinkle in his eye” and, somewhat patronizingly, referred to Tilden as the “merry little woman” subdirector. Reflecting on the laboratory space, he pointed out that it had all the standard tables, microscopes, reagents, books, and other laboratory apparatus equivalent to those in colleges and universities—except, he noted, its simple furniture had been built on site. Indeed, roughing it was part of the sense of wilderness captured by many of the photographs. (Fig. 12) Most participants expressed the spirit of adventure that heightened the scale and drama of the coastal site, from gigantic cedar trees and hollow coastal caves to adventuresome hiking and camping in largely unexplored territory.

Although the station provided a seemingly unlimited wealth of specimens each year, the enrollment drifted downward, and it attracted almost no Canadians or researchers from other major universities.^83^ In 1905 attendance dropped to fifteen. The 1906 session was overcast by the fact that Conway MacMillan had resigned from the University and his attendance marked the end of his affiliation with the station as well. Some attributed his departure to differences with the Board of Regents, which informed him that he was to return all apparatus, books, or other property taken to the station and to cease using the name of the university on any advertisements.^84^ However, it was also clear that MacMillan had begun speaking to business groups about effective marketing and making plans to leave for a new and better paying career.^85^ Indeed, shortly thereafter he left botany completely to accept a position in advertising in Philadelphia after attending his final session at botanical beach.^86^

Nonetheless, the 1906 session was again well attended and recorded both in photographs and a series of anecdotal stories in Sunday issues of the *Minneapolis Tribune* by undergraduate Emily Crosby.^87^ At the final dinner, as Postelians packed to go home, MacMillan read appreciative notes from former participants as current attendees dressed up again in their “civilized clothes.” The impending return home caused one student to comment, ruefully, “My skirt felt so queer. I was stepping on it or kicking it up all the time.”^88^ Her comment underscored the distinctive quality of the Minnesota station and the feeling of liberation enjoyed by the women who attended. Once again, Tilden and MacMillan coordinated a volume of *Postelsia* intended to commemorate the 1906 session.^89^

## Establishing an Independent Career

Tilden posted an invitation for the seventh season, 1907, on her own and attracted twenty-five attendees who convened at the Hotel Dominion for what turned out to be the final six-week session. Her brief advertisement described the station as a “biological camp and laboratory.” She highlighted the distinctive location, noting, “The combination of sea and forest and the absence of any distractions of the town make this camp one of the best spots in the country for study, recreation and health.” Moreover, July and August had the sunniest weather on that western coast, with an absence of noxious mosquitoes. Another advantage, she pointed out, was the lack of restrictions on legitimate collecting; previous visitors had brought back large amounts of botanical and zoological material.^90^ The session again attracted a significant number of high school and normal school instructors.

Now fully in charge of the Minnesota Seaside Station, Tilden opted to redesign the curriculum to include a sustained study of designated sites. She may have been influenced by a systematic quadrant method used by the new head of Minnesota’s Biology Department, another Bessey student, ecologist Frederick Clements (Hagen 1993). She designated twelve different plots on the coastal ledge, assigning students to devote four hours each day to the study of their assigned plots and record the marine life changes effected by tides, time of day, and other factors.^91^ At the end of the session, participants reported enthusiastically about their experience to a reporter in Victoria. Tilden herself reflected that it was “the best class we have ever had.”^92^

This session also recruited two Detroit area high school teachers who subsequently joined “Joe” Tilden on her first trip to Tahiti. Winifred B. Chase (1877–1959) had a degree in botany from the University of Michigan in 1903 and her close friend, Bernice Leland (1882-?), had an education degree from the University of Pennsylvania. Their shared curiosity and commitment to field studies led them to the Minnesota Seaside Station in 1907 (Jones 1966).^93^ Although working intensively, they found time for fun and teasing, and a fellow student penned this doggerel about Winifred:

An athletic young woman named Chase

Who was always first in the race

Put a log on her shoulder

Stepped from boulder to boulder

And said, “I can keep up the pace." (Jones 1966, p. 188)

The easy camaraderie of women at the station suggests the homosocial linkages identified by Carol Smith-Rosenberg in nineteenth century settings where close relationships were accepted, even anticipated.^94^ Informal settings set at a distance from social conventions provided space to live more freely. Not surprisingly, a network of Tilden’s friends, like Caroline Crosby, and relatives including her mother and maternal aunt found the station a congenial space to both work and explore the natural world around them. Their collegiality extended to the women who joined each year.

However, despite positive reviews, the successful 1907 session turned out to be the last summer school. Although *Science* announced a 1909 session, it was never held. Other stations were also announced in that issue, including one on Orcas Island led by R. K. Beattie of the State College in Pullman, Washington. Privately, Tilden explained to a potential attendee the circumstances that undergirded her decision to cancel the 1909 session: business would keep MacMillan from attending, her book *Minnesota Algae* was just reaching completion, and the new department chair, Frederic Clements, was not supportive.^95^ So, to the disappointment of likely enrollees, there would be no summer school that year. In a manuscript chronology, she reported that she did not want to maintain that “loss of personal time, energy, and money.”^96^ Tilden seems to have never returned to botanical beach, although she kept alive a hope that a summer program at the station might be re-established.^97^ Without university financial support and with Minnesota colleagues drawn to the more conveniently located Itasca Field Station at the headwaters of the Mississippi River, fully established in 1909, she was free to move on.^98^

In fact, Tilden had already begun to turn her attention elsewhere, seeking adventure and research opportunities further across the Pacific, where she investigated algae in the French Polynesian Society Islands, especially Tahiti, and then in New Zealand in the fall and winter of 1909–1910.^99^ Her completed book, *Minnesota Algae*, brought her promotion to full professor in 1910. While on leave in Tahiti, she obtained land for a small house used as a research base near Papeete before returning to her academic life in Minnesota the following spring. This next stage of her career became particularly productive because Tilden caught the wave of what Philip Rehbock noted as a particularly Pacific moment in the sciences after World War I, one marked by the intensifying economic and diplomatic imperialism of the United States in the South Pacific (Rehbock 1988; Capozzola 2020). Tilden made at least eight excursions, one as far as Western Australia, before her retirement in the mid-1930s, and she actively participated in the Pan-Pacific Congresses of the 1920s.

## Conclusion

The Minnesota Seaside Station was a distinctive field laboratory, coordinated by two faculty members. One was an intrepid woman whose botanical expertise was central to the program and whose organizational skills enabled implementation. The other was an ambitious and articulate spokesperson keen to make his own professional mark in line with that of his expanding university. Both envisioned a fresh model for a research station and encouraged a casualness and even unconventionality among those who attended. They positioned their site among East Coast seaside laboratories even as they marked theirs as distinctive. The Minnesota Seaside Laboratory focused on botany, especially algae and lichens, but included classes and faculty who specialized in zoology and geology with attention to ecology. Tilden encouraged women graduate students and teachers to attend, and MacMillan emphasized the inclusiveness of the station in his publications. In retrospect, the dedicated founders were perhaps naïve about the challenges of a remote site far from their home base, but its innovative strategies for living and working on the rugged west side of Vancouver Island provided research opportunities for women interested in marine science that were rare in the early twentieth century. The station, built by faculty at a public Midwestern university, reflected a turn of the century moment when that region sought to assert itself as both significant and distinctive (Bain 1916; Larson 2007).^100^ The land grant universities, operating without much philanthropic support and increasingly dependent on fees and tuition, used such sites among their strategies to pursue research in the early twentieth century.

The station was short-lived but hardly a failure. Tilden proudly reported 35 publications on algae alone and nearly 90 total publications that resulted from those seven years on site. The roughly 200 participants included teachers who gathered nature study specimens for teaching, graduate students collecting research materials for advanced degrees, and academic faculty whose research resulted in publications. One instructor in the early years, Francis Ramaley (1870–1942), found the experience inspirational, and he went on to establish the University of Colorado’s Mountain Laboratory in Tolland, Colorado, known for its ecological orientation (Vetter 2012).

There was no single reason why the Minnesota Seaside Station proved unsustainable. Local historians pointed to the conditions for travel and supplies, including a primitive trail with log bridges over crevasses and rocks slippery with damp mosses and lichens.^101^ Tilden blamed the University for failing to provide financial support. Others noted the distance from the organizers’ home base, the lack of a philanthropic sponsor, and even the difficulty of Midwestern management at a remote Canadian site. No accounts mention possibly competitive projects, including one at Nanaimo and the emerging University of Washington’s Puget Sound Biological Station (Needler 1958). These Northwest regional initiatives, not intended as alternatives to the Minnesota Seaside Station, would likely have limited interest in the less accessible station on the distant ocean side of Vancouver Island.^102^ Tilden clearly held no resentment toward the other initiatives and taught at the Puget Sound station in the summers of 1915 and 1917 (Hansen 2018, p. 190).^103^

Tilden emerged from her work at the Minnesota Seaside Station with new skills and confidence. She remained among a minority of women who found their way into university positions at the turn of the century, but they remained a distinct minority (Rossiter 1982). Having a mentor who provided sustained support could be essential for their success. For at least a decade, MacMillan and Tilden enjoyed something like the “creative partnership” of dual career couples where gender-shaped responsibilities provided opportunities but also obscured achievements (Pycior et al. 1996; Lykknes et al. 2021). Theirs was a professional collaboration, each supported by other personal relationships. MacMillan was married with two children and Tilden had strong female friendships. At the same time, a few fragments of correspondence suggest they maintained contact even after MacMillan left for a marketing position in Philadelphia.^104^ In certain ways, Tilden was quietly diffident, largely moving without comment past the normalized masculine behaviors that were default in the building of the station and on campus. Mutually dedicated to establishing the station and their department, the two scientists absorbed and reflected visibly gendered roles that were not rigid and could be negotiated, sometimes playfully, as when men donned female costumes in play acting and when young women pragmatically dressed in overalls for inland hikes. Tilden and MacMillan assumed roles that were complementary rather than co-equal. In many ways, their engagement in research and familiar camaraderie seems to have provided a model for those who joined them on Vancouver Island.

MacMillan served as a mentor for Tilden when he encouraged her botanical research and explicitly facilitated her appointments to the survey and then to the faculty. At the same time, he presumed she would take on service responsibilities, maintaining the department library and assisting him and other colleagues with their illustrations.^105^ Deference and patterns of authority established while she was a student persisted even as she established her own reputation and independent publications, apparently ignoring those who casually mentioned her as “assistant” or “secretary” to MacMillan.^106^ While media publicity about the station may have obscured her role, she was, as this article has shown, central to its establishment and operation.^107^ MacMillan’s ongoing support reflected his respect for her work. She, in turn, looked to him for advice and affirmation in developing her own research program and teaching.^108^

Historians continue to explore the complex gendered relationships that could both constrain and empower women’s opportunities in science. Recent research emphasizes that the distribution of tasks was rarely simplistically binary (masculine/feminine). Tilden and MacMillan reflected but were not confined to gender roles that they, to use the observation of Erika Milam and Robert Nye, simultaneously adapted and also resisted (Milam and Nye 2015).^109^ MacMillan was perhaps uncomfortably framed by masculine norms and the pressure to find a more financially substantial and manly occupation when he left the university for a better paying advertising career in 1906.^110^ A colleague once suggested he had “a mind like lightening and a tongue like a whiplash,” which may have operated to limit his success in business.^111^ His resignation released Tilden from some subsidiary responsibilities undertaken while he was department chair. By then, his early mentorship, although qualified, and the Minnesota Seaside Station experience had given a working class young woman the experience and confidence to continue her studies of Pacific algae as she organized expeditions to Tahiti and beyond, reaching as far as Western Australia by the mid-1930s.^112^ There, too, Tilden recognized the value of making connections with local settler and indigenous residents.

This essay adds to the literature on the history of women in science by demonstrating that successful careers for a limited number of academic women at the turn of the century were built on considerable negotiation, some degree of assertiveness, and often strategic planning to find a mentor who recognized—and in some cases took advantage of—a protégé’s skills. Josephine Tilden’s subsequent career demonstrates that the expertise and informal leadership developed under the qualified mentorship of MacMillan at the Minnesota Seaside Station served her well for achieving a tenured position and coordinating future research expeditions.

## Footnotes

1 *Minnesota Chats* 19 (April 29, 1937): 1, 3–4. A campus reporter interviewed Tilden as she moved toward retirement, and the Minnesota Seaside Station remained one of her clearest and happiest memories.

2 Notices of the Faculty Women’s Club are in the Josephine Tilden Papers, Box 1, Folder 1, University of Minnesota Archives (UMNA).

3 Rossiter and others have documented the importance of a critical number of women in a field as a factor in the success of the individuals involved.

4 Some accounts of Tilden essentially omit MacMillan’s role at the station and emphasize her role with presumptive explanations, perhaps the result of the paucity of personal records. See, for example, Haley Healey (2020). This perspective is an important corrective to and striking reversal of contemporary accounts by male colleagues that gave most, if not full credit to MacMillan. Historical perspectives change, and this essay seeks to identify the dynamic balance of responsibility and credit, while acknowledging the factor of gender bias.

5 Tilden Genealogical Book, Tilden Papers; see also Tilden Information File, both in UMNA.

6 Into the 1880s, various faculty members taught aspects of natural science until separate departments of geology, botany, and zoology were established. *Biennial Report to the Board of Regents of the University of Minnesota for 1881 and 1882* (Minneapolis, 1883), p. 6.

7 MacMillan to Nathaniel Britton, September 26, 1890, Britton Papers, NYBG.

8 The UMN *Gopher Yearbook* for 1896 (p. 46) indicates Tilden was active in the Botanical Society and Fortnightly Scientific Society.

9 Botanical Notebook, Sophomore Year, January to July 1893, Tilden Papers, UMNA. She published six reports over five years related to that summer’s intensive research.

10 An anonymous reviewer helpfully pointed out the article with MacMillan’s assertion. Henry Frances Nachtrieb, a colleague in zoology and advocate for Simplified Spelling, reflected to R. T. Young, “We had a sort of survey hedquarters [sic] on Gull Lake one summer but never establishtd what could be cald a station.” December 9, 1911, Nachtrieb Papers, UMNA.

11 Other young professionalizing botanists similarly raised funds, including Edith and Frederic Clements as they financed their Alpine Laboratory.

12 Tilden inadvertently found herself in a kind of competition with Francis Collins, an active bryologist in Malden, Massachusetts. See Tilden’s letters to Collins, Collins Papers, American Philosophical Society.

13 This first of her bound exsiccatae, produced in 1894, became Century I of Tilden’s seven volume series of *American Algae;* her later explorations produced three additional volumes of *South Pacific Algae* and two of *South Pacific Plants*. Each Century issue had one hundred specimens, dried and on paper with formal identifications and locations of origin for each specimen. Her advertising circulars went to over a hundred herbaria, universities, botanical gardens, and other institutions in North America and Europe; she sold more than two dozen Century volumes at prices ranging from $6.50 to $10. Tilden Papers, Notebook 1897–1898, UMNA.

14 The buyers included individuals and institutions ranging from Harvard, Berkeley, and public gardens in New York and St. Louis, to several institutions in Britain, Germany, and Australia. Tilden Papers, Notebook 1896–1898, Box 3, UMNA.

15 Running from Minneapolis to the Pacific Coast, the railroad began service to Yellowstone National Park in 1883 and quickly increased scientific interest among natural scientists.

16 Tilden’s mother served as chaperone and companion for her only daughter and youngest child and assisted her in the botany library on campus. The gender norms constraining women who engaged in scientific travel were loosening in this period; for examples, see Adams (2010).

17 This practice was relatively common because individually printing identification cards locally was time-consuming and expensive.

18 New England algae expert Frank S. Collins observed in the 1890s, “One has to scratch pretty close to find new marine algae in New England now,” quoted Hallender. *The Wild Garden*, n 7, p. 54.

19 James Pringle documents the intermittent and piecemeal studies of Vancouver Island until well into the twentieth century (Pringle 1995).

20 Based on an oral interview with someone who knew Tilden, Hallender suggests that Tilden’s plan was to go to the British Museum for a Ph.D., but museums did not give advanced degrees. See Hallender 1991, p. 55; also see manuscript chronology by Tilden in Tilden Information File, UMNA.

21 At some point in the 1880s, the son apparently changed the spelling of his last name. *The Hillsdale College Catalog for 1870* (pp. 4 and 6) indicates his father, George McMillan, was librarian as well as professor of Greek and Latin.

22 Various sources suggest that MacMillan briefly spent time at Harvard after leaving Nebraska, some mentioning 1887–1888, but I have not been able to confirm any study there. See Humphrey (1961, pp. 159–160). Humphrey’s collection of short biographies includes just two women, Jane Colden and Elizabeth Britton.

23 His relationship with William K, Brooks at Hopkins seems fraught. MacMillan issued a strong critique of the way in which botany, in relationship to zoology, was marginalized at Johns Hopkins and Columbia in “On the Emergence of a Sham Biology in America,” (1893a, b). An almost immediate rebuttal came from a recent Hopkins Ph.D., Francis H. Herrick, “On the Teaching of Biology” (1893). Three years later MacMillan felt vindicated when the University of Chicago and Columbia renamed biology departments to offer distinct programs in zoology and botany and wrote “On the Disappearance of Sham Biology” (1896). The final word seems to have been from Brooks chiding MacMillan for inaccurately presuming that Hopkins offered a Ph.D. in biology rather than a more specific science. See Brooks, “Zoology and Biology” (1896). MacMillan seemed intent on defending botanical studies as related to the new biology and using the methods of physiology.

24 MacMillan would have known of the Hopkins Marine Laboratory in Beaufort that operated from 1880 to1886.

25 See a series of letters to Britton between 1890 and 1892 in the Mertz Library of the New York Botanical Garden. The publication of *Botanical Studies* had not been explicitly authorized by the Regents and fed a growing resentment of the expenditures by the independent-minded young faculty member.

26 C. A. Ballard to Dean E. M. Freeman, n.d. [1934], MacMillan Information File, UMNA.

27 Among multiple activities, he organized a Central Botanists Association for Midwesterners and coordinated the local Grand Army of the Republic program, covered in the *Minneapolis Journal,* January 6, 1903 and May 25, 1904.

28 Judge William C. Leary to E. M. Freeman, Dean of the College of Agriculture, May 14, 1934, MacMillan Informational Folder, UMN.

29 Among numerous examples, see “Botanists to Meet,” *Minneapolis Journal* (December 27, 1904): 7. It was the second annual meeting of the regional group.

30 Classic accounts like Manning constructed these tensions as federal versus state authority, certainly an accurate assessment, but they overlook the regional tensions that were magnified in the period.

31 Tilden struggled financially throughout her life, typically overly optimistic about how to fund her projects. She died essentially destitute. On the double challenges of class and gender, see Samantha Muka (2014a, b).

32 C. Otto Rosendahl, who had started coming to the Minnesota Seaside Station as an undergraduate in 1901, offers a comparison with Tilden. When he completed his degree in 1905, he was encouraged to study in Berlin, where he took his Ph.D. He returned to a faculty position and in following decades served intermittently as chair of the department. Gladys Crowther, “Forty-one Years on the Faculty,” Rosendahl Information File, UMNA.

33 Seafarers best knew the west coast waters of Vancouver Island, but Tilden wanted to approach the littoral space from shore, seeking to understand the plants and animals that inhabited it. See von Hardenberg 2021 and Nicholson (1966).

34 Tilden Papers, Travel Notebook 1898, UMNA. This notebook and the notebook for 1896–1898 detail travel expenses and chemical preparations for various species and other basic laboratory records. She left Victoria with five boxes of botanical specimens in glass jars and two boxes of specimens in tin cans along with various equipment used for mounting and preserving them.

35 Tilden’s small travel notebooks indicate that she relied on Admiralty Charts to predict tidal action, which allowed her to time her trip to coincide with an unusually low tide and a maximum exposure of algae.

36 On collegiate museums and with special reference to the one at the University of Minnesota, see Kohlstedt (2017).

37 There is no record of a sale, but Tilden did gain title and paid minimal taxes on the land for three decades. Baird represented the provincial government eager to encourage settlement, and he apparently could authorize small land grants to someone intending to build on the property.

38 *Plant World,* initiated in 1898 by F. H. Knowlton of the U.S. Geological Survey, was published until 1919 when it became part of *Ecology*. It replaced the short-lived *Asa Gray Bulletin* and was sponsored by the Wild Flower Preservation Society and identified as “a monthly journal of popular botany.”

39 Tilden (1898), quotation on 148. Her style here was similar to that of several British women writers who also found inspiration in algae; see Gates (2002, esp. pp. 558–567).

40 In the second volume of *Plant World*, her essay, “The Study of Algae in High Schools” advocated for the scientific and practical benefits of botanical education and provided a quick classification guide for algae (Tilden 1899). Tilden offered to send specimens to interested teachers, referred to as “she” in the text.

41 Tilden detailed her travel and research in the first journal (of just two) of the seaside station named *Postensia* (Tilden 1901). Crosby was an heir to the Washburn-Crosby Mills, which eventually became part of General Mills; she frequently collected with Tilden and joined her on several of her longer expeditions.

42 On the Western ambitions and engagement in the Pacific, see van Dijk (2015).

43 Albert G. Mayer observed that such stable locations were the future of biological research, replacing the large expeditions that had characterized earlier centuries, although he still made highly focused excursions himself (Stephens 1997). On a particularly productive, ecological project in Illinois, see Schneider (2000).

44 Few of these new universities had resources for research aside from some state funds for surveys and the Hatch Act of 1887 that undergird agricultural investigations. Private initiatives provided some resources; in Wisconsin, the son of a wealthy industrialist, zoologist Edward P. Allis, created Allis Lake Laboratory and hired Charles Whitman to manage it from 1887 to 1889 (Richter 1957).

45 Alfred H. Sturtevant apparently liked to comment, “Marriages are made in heaven but there is a branch office in Woods Hole.” Quoted in Richmond 2012, p. 150. I thank Don Optiz for reminding me of this reference.

46 The *New York Times* also mentioned a temporary research station in Port Townsend operated by faculty from Columbia College [University] who worked “in line with” the U.S. Fish Commission Service, but there is no evidence it was sustained (Anon. 1896).

47 The Tortugas Marine Laboratory was similarly remote but had practical amenities and equipment, provided by the Carnegie Institution for Research, which paid the scientists who used that facility off the Florida Keys (Colin 1980).

48 An ambitious plan to build a six foot wide corduroy road from Port Renfrew to the botanical beach was approved by the British Columbia Parliament (courtesy of the members from Esquimalt and San Juan), but after a short initial start the road was never completed (MacMillan 1902a, b).

49 The actual time at the station that first year was just four weeks because the Minnesota group took essentially a week for the train trip with several stops along both ways. In subsequent years, travel times were shorter (Anon. 1901). 

50 The Minneapolis *Star Tribune* announced that the University of Minnesota was sponsoring a seaside station on Vancouver Island, but MacMillan was quick to write a corrective indicating that the project was a faculty initiative. Minneapolis *Star Tribune* (January 21, 1901), p. 5. He acknowledged theirs was a modest enterprise, a camp on “a shore extraordinarily favorable for marine investigations.” The founders hoped to make clear its viability before asking the Regents to take responsibility for it. The leaders had permission to borrow microscopes and books from the university to take to the station. A Minnesota colleague, Henry Nachtrieb, reflected that it was “a private enterprise from first to last.” Nachtrieb to R. T. Young, December 9, 1911, Nachtrieb Papers, UMNA.

51 It remains unclear who paid for these rather basic buildings. Tilden mentions help from the Canadian government and the Soo Line, which provided a link between Minneapolis and the Canadian Pacific Railway (Anon 1937, p. 4). The buildings were simple, constructed with logs taken on site; the larger building foundation was 60 × 25 feet and the smaller 25 × 12 feet. Some anecdotal accounts suggest (without evidence) that Tilden provided the funding; however, her family was working class, and she was persistently troubled by money problems. Citing an undocumented letter, Scott (1974, p. 34) states that Tilden told someone local that she and MacMillan used their own salaries to maintain the property. A newspaper suggested only that money was raised by “private subscription.” *Minneapolis Journal* (January 26, 1901), Part I: 1.

52 Typescript of R. Bruce Scott, “Botanical Beach,” Minnesota Seaside Station Information File, UMNA.

53 Caswell Ballard further commented the MacMillan had a “thoroughly striking personality coupled with initiative and enthusiasm” and was the kind of teacher who “aroused deep interest in nature and gave me confidence in my own ability.” C. A. Ballard to Dean E. M. Freeman, n.d. [1934], MacMillan Information File, UMNA.

54 Tilden Papers, Travel Notebook, 1901–104, Box 3, UMNA.

55 Rebecca Toov, “Attendees of the Minnesota Seaside, 1901” is a typescript compilation of the attendees from 1901 to 1907. Minnesota Seaside Station Information File, UMNA.

56 See Otto Rosendahl’s Photograph Book is in the Department of Botany Papers, Box 33, Folder 370, UMNA.

57 Hibbard’s photographs from the trip are integrated into the Department of Botany Photographs, UMNA.

58 The Midwesterners were open-minded and interested in their encounters with Asian peoples. There is no commentary about this Chinese cook aside from that of his welcomed culinary skills. Their Japanese participant, Kichisaburo Yendo, encouraged cultural exchange by sharing his kimono robes and fully participated as a scientific colleague in the identification of algae and its uses in Asia.

59 These came from the Wachter Seafood Products Company when it went out of business in 2020, donated to UMNA by Carrie Minucianni, along with reprints, some books, student dissertations, and a few copies of Century materials. Unfortunately, almost no Tilden or MacMillan correspondence surfaced.

60 Tilden Papers, Travel Notebook for 1898 [with notes for subsequent trips], Box 3, UMNA.

61 Perhaps being single could be an advantage, because both in terms of legal status and cultural norms married women—as contemporary feminists pointed out—often found themselves constrained in financial matters and mired in domestic tasks. For examples, see Rossiter (1982) and Tonn (2019).

62 Tilden Papers, Black Notebook, p. 8, UMNA.

63 Takehito Hiraga kindly confirmed that this was Kichisaburo Yendo, then a graduate student at the Imperial University at Tokyo, who subsequently taught at Haikkido University. He notably discovered *Corallina vancourveriensis* while at the station and shared specimens with colleagues in Europe as well as in the United States. See http://sourui.org/publications/sorui/list/Sourui_PDF/Sourui-62-02-00a.pdf. Hiraga to S. G. Kohlstedt, 21 January 2021. Tilden’s Travel Notebook, 1898 (but with entries for Hawaii in 1900) mentions Yendo, so probably she met him there and invited him to the station.

64 His paper “Uses of Marine Algae in Japan” introduced the group to economic algae, and it included lovely foldout illustrations of Japanese algae used as frontispieces to *Postelsia* 1901. This may have inspired Tilden to introduce “industrial botany” into the curriculum with an emphasis on algae as a food source.

65 Alice Misz to Mother, 23 July 1906, Misz Papers, UMNA.

66 Alice Misz to Mother, 23 July 1906, Misz Papers, UMNA. Misz had recently graduated, learned about potential teaching positions, and got informal career advice from more experienced participants at the station.

67 The course on Problems in Algology was open to graduate students and listed Tilden as Assistant Professor, with the additional comment that “Special facilities for study are offered by the Minnesota Seaside station on Vancouver Island, which is open during the summer vacation.” University of Minnesota *Catalogue for 1907–1908*, p. 126.

68 The term *nature study* was in popular use in this period, often a term for informal engagement. It also named a quite specific curriculum in public elementary schools, making the Minnesota station attractive to normal school instructors as well as high school and other teachers (Kohlstedt 2010).

69 The first *Postelsia*, apparently published in 1902, sold for $3.25, including shipping. There were 250 copies for purchase, and a few went out for review.

70 Participants named the main building Sea Palms, while the zoology building became the Formalose Club.

71 Misz to Mama, 30 July 1906, Misz Papers UMNA.

72 Moore and Toov, “Minnesota Seaside Station.”

73 Thus, for example, Tilden brought back the daughter of a Tahitian leader to St. Paul, where Ina Salmon attended school and learned English in the 1910s. Tilden also wrote a draft history of the people of the islands. Tilden Papers, UMNA.

74 Tilden Papers, Record Notebook, pp. 157–160, UMNA.

75 Tilden to MacMillan, n.d. [1910s], Tilden Papers, Box 2, Folder 1901–1927, UMNA.

76 Colleagues frequently credited her with collecting for them in articles in *Minnesota Botanical Studies.* See MacMillan (1898–1902).

77 Charles Bessey compiled “Botanical Notes” regularly for *Science* and included the notice in a section subtitled “Pacific Seaside Botany” *Science* 15 (Bessey 1902). Noting the difference in resources, Bessey nonetheless concluded that both Pacific projects were “excellent stations for study and research.”.

78 Tilden Papers, Travel Notebook C, pp 4–5, Box 3, UMNA.

79 Tilden Papers, Record Notebook, pp. 157–160, UMNA.

80 Conway MacMillan, letter to the Editor, *Science* N.S. 18 (July 10, 1903): 57–58.

81 MacMillan described his new venture in annual scientific meetings and circulated flyers widely.

82 The organizers had larger ambitions, too, for attaining a steam launch to explore further offshore as well as a system of water pipes that might allow for more laboratory research (MacMillan 1902c).

83 While Canadian participants were encouraged, very few ever came, perhaps because the University of British Columbia was not established until 1908 so there was no nearby pool of faculty and students seeking a research experience.

84 Board of Regents, Minutes of 2 May 1907, pp. 48–49. The minutes also note that, although paid, MacMillan had not yet submitted his final report on the Natural History Survey. Later a still frustrated Tilden wrote about the abandonment (from her perspective) of a promise from the administration to take over responsibility for the station. Her recollection is challenged by a contemporary newspaper account provided by MacMillan in 1901. Letter quoted in Pelley (1985, p. 6).

85 “Factors in Modern Advertising,” *Minneapolis Journal* (2 February 1906): 6; and “Is Minnesota Generous to Her Great State University?” *Minneapolis Journal* (June 1906): 11.

86 The local press covered Macmillan’s departure from the University, including his well-attended farewell party at the Commercial Club and his reportedly lucrative offer in Philadelphia. See “Farewell to MacMillan,” *Minneapolis Journal* (28 June 1906): 6. His doctoral students included Francis Ramaley of the University of Colorado, Charles M Fraser of the University of British Columbia, Robert F. Griggs of George Washington University, and Harold L. Lyon of Honolulu, Hawaii.

87 Emily Crosby published nine chatty articles in the *Minneapolis Tribune* between 1 July and 26 August 1906.

88 Misz to Mama, 8 August 1906. Misz Papers, UMNA.

89 Charles Bessey positively reviewed the volume of essays in *Science* 25 (11 January 1907): 62–63. Most of the volume reflected studies done at the station, with a long paper on plant (primarily tree) distribution by Minnesota’s Otto Rosendahl.

90 Josephine E. Tilden, Minnesota Seaside Station, Seventh Annual Season [Flyer for 1907]. No mention was made of academic credit, simply the opportunity to “get a very complete introduction to a knowledge of the plants and animals of the shore.”.

91 Tilden taught algae, Ned Huff lectured on nature study, Arthur Schneider (University of California) on lichens and bacteria, and Oscar Oestlund on entomology and plant lice. Another class member told the press it was “the most enjoyable time we have ever had.” *Victoria Daily Colonist* (20 August 1907), p. 16. Carl Rosendahl and Arthur Johnson had walked to the station from Duncan and Aberni on the eastern side of Vancouver Island to study inland plant life along the way.

92 *Victoria Daily Colonist* (August 20, 1907), p. 16.

93 Both Chase and Leland taught high school in Delray, now part of Detroit, and continued botanizing in Michigan and further afield in coming decades. Chase took an MA in botany in 1915 and became an instructor in what would become Wayne State University while Leland taught at the Detroit Psychological Clinic.

94 Carroll Smith Rosenberg utilized this term in a path-breaking essay (1975). Leland wrote a memoir of Chase that emphasized her activism on behalf of women, including serving as advisor to women at Wayne State University and establishing a loan fund for them. See the typescript in the Winifred Chase Papers, WSUA.

95 Tilden typically conducted field research throughout Minnesota in the late spring and early fall, before and after the trips to Vancouver Island. The book summarized her nearly twenty years of local investigation that had been sandwiched between teaching and more distant travels.

96 “Notes and News,” *Science*, 39: 822. Tilden to Bernice Leland, 6 June 1909, Leland Papers, Box 1, Folder 3, UOSC.

97 Her colleagues Otto Rosendahl and Frederick Butters did, however, go back in the summer of 1910 and found the site overgrown (Abbe 1948; Rosendahl Information File). In 1913, Tilden again asked the university regents to sponsor the station and apparently wrote to other universities offering the site, but without success; reported by Richard Norris to A.C. Bowdish, Jan. 3, 1955, Department of Botany, Box 11, Tilden folder, 1931–1967.

98 The local stations intended for long term studies were, in many ways, the antecedents of Long Term Research Centers (LTER) sponsored by the National Science Foundation (Rumore 2008). The Iowa Research Center, founded in 1911, is another example of university consortium education (Lannoo 1996).

99 Tilden Papers, Field Notebook D, 1909–1913, Box 3, UMNA. Tilden took her mother, aunt (Mrs. Matthew Perrott), Leland, and Chase with her to Tahiti from September to November 1909 and then went on to New Zealand before returning to Tahiti. She subsequently took at least four more research trips through the Pacific, including sabbaticals in 1912–1913 and 1934–1935. Her mother died in 1919, a loss of Josephine Tilden’s staunch supporter.

100 Newton Horace Winchell, for example, deliberately launched *American Geologist* in 1888 to advance state surveys and the Midwest in response to William Powell’s increasingly powerful, nationally focused U.S. Geological Survey.

101 Scott (1974) also reported there was increasingly irregular steamer service along the coast that constrained development.

102 The site is now part of Juan de Fuca Provincial Park, and its botanical beach continues to be a research site for both Canadians and Americans.

103 T.C. Frye to Tilden August 5, 1914, described the Friday Harbor laboratory and expected to work with her there the following summer if she did not go to Tahiti. Tilden Papers, Box 1, Folder 1, UMNA.

104 About the time MacMillan left Minnesota in 1906, his wife, formerly Maude R. Sanborn, and daughters moved to Portland, Oregon. Letter from his sister, Bertha McMillan, to Dean E. M. Freeman, 29 April 1934, MacMillan Information File, UMNA.

105 MacMillan’s typescript, “Observations on the Fenestrations of Martensia.” MacMillan acknowledged Hawaiian specimens from Tilden on which he based his analysis. Folder identified as Undated [after 1901], Box 1, Tilden Papers, UMNA.

106 Several examples point to the “able assistance” of Tilden to MacMillan, including his sister Bertha’s suggestion that Tilden “acted as his secretary for many years.” Typescript biographical sketch and miscellaneous items in MacMillan Information File, UMNA.

107 Biographical accounts of MacMillan often give him complete credit for the station. See, for example, his biographical entry: Natural History Museum, London, JStor Global Plants: https://plants.jstor.org/stable/10.5555/al.ap.person.bm000152508.

108 In an undated fragment copy of a letter [probably mid 1910s] to MacMillan, Tilden reflected on her financial problems and wished she had “your good advice as I used to have it.” Tilden Papers, Box 2, MacMillan Folder, UMNA.

109 Erika Lorraine Milam's and Robert A. Nye's thoughtful introduction to *Osiris* (2015) examines the complexity of gender characterizations. Articles by Michael Robinson (2015) and Michael Reidy (2015) reflect on the style of masculinity that seemed to intensify in outdoor and exploration projects in this period and that echoed in the assertive style of MacMillan.

110 Other factors probably included MacMillan’s failing marriage and his alienation of the university governing board. In an undated letter she may or may not have mailed, Tilden implied that alcohol and gambling limited MacMillan’s success after he left. Expectations for men as well as women faculty could be overwhelming, but the only recorded explanation for his departure was his intention to earn a higher salary.

111 This undated clipping gives Otto Rosendahl as the commentator. Rosendahl Informational File, UMNA.

112 Tilden retired in 1937, when she reached mandatory retirement age, and she received permission to take books and much of her collection with her to Florida for ongoing work.
